# Ras GTPase Activating Protein CoIra1 Is Involved in Infection-Related Morphogenesis by Regulating cAMP and MAPK Signaling Pathways through CoRas2 in *Colletotrichum orbiculare*

**DOI:** 10.1371/journal.pone.0109045

**Published:** 2014-10-02

**Authors:** Ken Harata, Yasuyuki Kubo

**Affiliations:** Laboratory of Plant Pathology, Graduate School of Life and Environmental Sciences, Kyoto Prefectural University, Kyoto, Japan; Zhejiang University, China

## Abstract

*Colletotrichum orbiculare* is the causative agent of anthracnose disease on cucurbitaceous plants. Several signaling pathways, including cAMP–PKA and mitogen-activating protein kinase (MAPK) pathways are involved in the infection-related morphogenesis and pathogenicity of *C. orbiculare*. However, upstream regulators of these pathways for this species remain unidentified. In this study, *CoIRA1*, encoding RAS GTPase activating protein, was identified by screening the *Agrobacterium tumefaciens*-mediated transformation (AtMT) mutant, which was defective in the pathogenesis of *C. orbiculare*. The *coira1* disrupted mutant showed an abnormal infection-related morphogenesis and attenuated pathogenesis. In *Saccharomyces cerevisiae*, Ira1/2 inactivates Ras1/2, which activates adenylate cyclase, leading to the synthesis of cAMP. Increase in the intracellular cAMP levels in *coira1* mutants and dominant active forms of *CoRAS2* introduced transformants indicated that CoIra1 regulates intracellular cAMP levels through CoRas2. Moreover, the phenotypic analysis of transformants that express dominant active form *CoRAS2* in the *comekk1* mutant or a dominant active form *CoMEKK1* in the *coras2* mutant indicated that CoRas2 regulates the MAPK CoMekk1–Cmk1 signaling pathway. The CoRas2 localization pattern in vegetative hyphae of the *coira1* mutant was similar to that of the wild-type, expressing a dominant active form of *RFP*–*CoRAS2*. Moreover, we demonstrated that bimolecular fluorescence complementation (BiFC) signals between CoIra1 and CoRas2 were detected in the plasma membrane of vegetative hyphae. Therefore, it is likely that CoIra1 negatively regulates CoRas2 in vegetative hyphae. Furthermore, cytological analysis of the localization of CoIraI and CoRas2 revealed the dynamic cellular localization of the proteins that leads to proper assembly of F-actin at appressorial pore required for successful penetration peg formation through the pore. Thus, our results indicated that CoIra1 is involved in infection-related morphogenesis and pathogenicity by proper regulation of cAMP and MAPK signaling pathways through CoRas2.

## Introduction

*Colletotrichum orbiculare* is the causative agent of cucumber anthracnose disease. The infection process is initiated by the recognition of an appropriate surface. A series of changes in the morphology upon recognizing the appropriate signals, including the formation of a specialized infection structure called appressorium, is important for the successful infection of the host plants.

Several signal-transduction related genes associated with these morphological changes have been characterized in *C. orbiculare* [1]. It also has been shown that the MAPK and cyclic AMP (cAMP) signaling pathways are linked to infection-related morphological changes in this fungus. A yeast MAPK kinase (MAPKK) kinase *STE11* homolog, *CoMEKK1*, is involved in the appressorium development in *C. orbiculare* [2]. *C. orbiculare* mutant with the adenylate cyclase encoding gene *cac1* shows defectiveness in conidial germination [3], however, no upstream regulators of cAMP and MAPK signaling pathways have been identified in *C. orbiculare.*

Ras is the prototypical member of the small GTP binding protein superfamily that plays a pivotal role in proliferation and differentiation in eukaryotic cells. In *Magnaporthe oryzae*, MoRas1 and MoRas2 interact with Mst50, which interacts directly with the mitogen-activated proteins Mst11 and Mst7 [4]. In *Fusarium graminearum*, FgRas2 is involved in hyphal growth and pathogenicity [5]. In *C*. *trifolii*, CtRas is involved in conidial germination and hyphal growth [6]. Ras acts as a molecular switch that exists both in the active (GTP-bound) and the inactive (GTPase-bound) states. Cycling between these two states is aided by the interaction of a GTPase activating protein and guanine-nucleotide exchange factors. In *Saccharomyces cerevisiae*, the Ras activity is controlled positively by the guanine nucleotide exchange factor (GEF) Cdc25 and negatively by the GTPase activating proteins Ira1 and Ira2 (GAPs) [7]. In *S. cerevisiae*, the RAS GTPase activating proteins Ira1 and Ira2 inactivate Ras1 and Ras2, which in turn activate adenylate cyclase, promoting the synthesis of cAMP from ATP. Ira1 and Ira2 are important factors for controlling cAMP production. The cAMP signaling pathway plays a pivotal role in transducing environmental cues during cell development in *Magnaporthe oryzae* [8]. Moreover, the cAMP signaling pathway of *C. orbiculare* plays a critical role in regulating conidial germination and pathogenicity [9]. Adenylate cyclase gene *MAC1* encodes a Ras-association domain [10], [11]. In terms of protein structure, the Ras protein putatively interacts with Mac1 in *M. oryzae*. It was shown that cyclase-associated protein Cap1 of *M. oryzae* directly interacts with Mac1 and plays a role in the activation of Mac1, which may function downstream of Ras [12]. In *Ustilago maydis*, *RAS2* promotes filamentous growth through a MAP kinase cascade, however, the function of *RAS2* in the cAMP signal transduction remains unknown [13]. Therefore, direct evidences for the involvement of the Ras protein in the cAMP signal transduction are not sufficient in phytopathogenic fungi.

Because Ras proteins transduce signals from the cell surface to various intracellular effectors, they are located and function only at the plasma membrane [14], [15]. Conversely, data provided by the yeast GFP fusion localization database [16] and other published data [17], [18] indicate that Ras2, Cyr1, Cdc25, and Ira proteins mainly localize at the internal membranes of the endoplasmic reticulum and mitochondrial membranes, but only marginally at the plasma membrane. Recently, it was reported that active Ras accumulates mainly in the plasma membrane and nucleus when the cells are grown on medium containing glucose, whereas it accumulates mainly in the mitochondria in glucose-starved cells [19]. Moreover, PKA activity causes Ira2 to move away from the mitochondria [19]. Therefore, it is considered that the behavior of Ras proteins and Ras GTPase activating proteins and their localization depends on various growth conditions. In plant pathogenic fungi, subcellular dynamics of Ras proteins and Ras GTPase activating proteins during infection and the location of this interaction remain unclear.

In this study, we identified a novel *S. cerevisiae* homolog gene *CoIRA1*, encoding the RAS GTPase-activating protein (RASGAP) of *C. orbiculare* and characterized the *CoIRA1* function in relation to the activation of cAMP-PKA and MAPK signaling pathways. By phenotypic analysis of the *coira1* mutant, cytological analysis of CoRas2 localization and BiFC assay, we showed that CoIra1 regulates these signaling pathways through CoRas2 as a negative regulator. Conclusively, we presented CoIra1 is involved in infection-related morphogenesis by regulating cAMP and MAPK signaling pathways through CoRas2 in *C. orbiculare.*

## Materials and Methods

### Fungal and bacterial strains

The 104-T (MAFF240422) *C. orbiculare* (Berk. & Mont.) Arx [syn. *C. lagenarium* (pass.); Ellis & Halst.] strain was used as the wild-type. All the *C. orbiculare* strains used in this study are listed in Table 1 and were cultured on PDA media (3.9% [w/v] PDA; Difco laboratories, Detroit) at 24°C. One shop TOP10 chemically competent *E. coli* cultured in Luria-Bartani media [20] at 37°C was used as a host for gene manipulation. When required, the supplement kanamycin was added to the medium at 50 µg/ml. *A. tumefaciens* C58C1 cultured in Luria-Bartani media at 28°C was used to transform *C. orbiculare* by AtMT.

**Table 1:** Fungal strains used in this study.

Strain	Description	References
WT	Wild-type strain of *Colletorichum orbiculare*	This study
Dl1–1	*coira* *1* disruptant of WT	This study
Dl1–2	*coira* *1* disruptant of WT	This study
El1–1	*CoIRA* *1* ectopic transformant WT	This study
Cl1–1	Dl1–1 complemented with *CoIRA* *1*	This study
Cl1–2	Dl1–2 complemented with *CoIRA* *1*	This study
DC1	*cac* *1* disruptant of WT	Yamauchi et al. 2004
DARS1	WT transformed with a dominant active form *CoRAS* *1*	This study
DARS2	WT transformed with a dominant active form *CoRAS* *2*	This study
iDNRA1	Dl1–1 transformed with a dominant negative form *CoRAS* *1*	This study
iDNRA2	Dl1–1 transformed with a dominant negative form *CoRAS* *2*	This study
DMK1	*comekk* *1* disruptant of WT	Sakaguchi et al. 2010
DRS2–1	*coras* *2* disruptant of WT	This study
DRS2–2	*coras* *2* disruptant of WT	This study
CRS2–1	DRS2–1 complemented with *CoRAS* *2*	This study
CRS2–2	DRS2–2 complemented with *CoRAS* *2*	This study
DRS2/DAMK1	DRS2 transformed with a dominant active form *CoMEKK* *1*	This study
DMK/DARS2	DMK1 transformed with a dominant negative form *CoRAS* *2*	This study
DRS2/RFP-RS2	DRS2 transformed with *RFP*-*CoRAS* *2*	This study
WT/RFP-RS2	WT transformed with *RFP*-*CoRAS* *2*	This study
WT/RFP-DARS2	WT transformed with RFP-*DACoRAS* *2*	This study
iRFP-RS2	Dl1–1 transformed with *RFP*-*CoRAS* *2*	This study
Vc-RS2	WT transformed with *VENUS*(1–158aa)-*CoRAS* *2*	This study
IRA1-Vn	WT transformed with *CoIRA* *1* -*VENUS*(159aa-238aa)	This study
Vc-RS2/IRA1-Vn	IRA-Vn transformed with *VENUS*(1–158aa)-*CoRAS* *2*	This study
IRA1-VENUS	WT transformed with *CoIRA* *1* -*VENUS*	This study
RFP-RS2/IRA1-VENUS	WT/RFP-RS2 transformed with *CoIRA* *1* -*VENUS*	This study
RFP-DARS2/IRA1-VENUS	WT/RFP-DARS2 transformed with *CoIRA* *1* -*VENUS*	This study
LA/IRA1-VENUS	IRA1-VENUS transformed with LifeAct-*RFP*	This study
WT/LA	WT with LifeAct-*RFP*	This study
iLA	Dl1–1 transformed with LifeAct-*RFP*	This study
DPS1	*pks* *1* disruptant of WT	Takano et al. 1995
DSD1	*ssd* *1* disruptant of WT	Tanaka et al. 2007

### Fungal transformation

For the fungal transformation, we used an AtMT protocol that was previously described [21] with slight modifications. The hygromycin-resistant transformants were selected on the PDA medium containing 100 µg/ml of hygromycin B (Wako Chemicals, Osaka, Japan), 50 µg/ml of cefotaxim (Wako Chemicals, Osaka, Japan), and 50 µg/ml of spectinomycin (Wako Chemicals, Osaka, Japan). The bialaphos-resistant transformants were selected on an SD medium containing 10 µg/mL of bialaphos (Meiji Seika Kaisha, Ltd., Tokyo, Japan), 100 µg/ml of cefotaxim, and 100 µg/ml of spectinomycin. The sulfonylurea-resistant transformants were selected on an SD medium containing 4 µg/ml of chlorimuron-ethyl (Chem Service West Chester, PA, USA.), 100 µg/ml of cefotaxim, and 100 µg/ml of spectinomycin.

### Genomic DNA blot analysis

The total DNA from the mycelia of *C. orbiculare* was isolated, and a DNA blot analysis was performed using a previously described method [22]. DNA digestion, gel electrophoresis, labeling of probes, and hybridization were performed according to the manufacturer’s instructions following standard methods [20]. DNA probes were labeled with DIG-dUTP using a BcaBESTTM DIG labeling kit (Takara Bio, Ohtsu, Japan). Hybridized DNA was detected with anti-Digoxygenin antibody Fab fragments conjugated to alkaline phosphatase (Roche Diagnostics, Tokyo, Japan). Light emission from the enzymatic dephosphorylation of the CDP-Star Detection Reagent (GE Healthcare, Tokyo, Japan) was detected using the Fujifilm LAS-1000 Plus Gel Documentation System (Fujifilm, Tokyo).

### Construction of the *CoIRA1* gene replacement vector and *CoIRA1* complementation vector

To replace the *CoIRA1* gene with the hygromycin-resistance gene, we constructed a *CoIRA1* gene-replacement vector pBIG4MRBrev-coira1. We first amplified the upstream region of the *CoIRA1* gene, the hygromycin-resistance gene, and the downstream region of the *CoIRA1* gene by PCR using the primer pairs CoIRA1F1A–CoIRA1R2D, CoIRA1hphF1C–CoIRA1hphR1D, and CoIRA1F2C–CoIRA1R1B, respectively. The primers used are listed in Table S1. Next, the pBIG4MRBrev–coira1 vector was constructed using the GeneART seamless cloning and assembly kit (Life Technologies, Carlsbad, California USA) with the amplified product and the *A. tumefaciens* binary vector pBIG4MRBrev. This vector, which contains a bialaphos resistance gene, was used as the gene replacement plasmid.

To perform a complementation assay of the *coira1* mutant, we constructed the CoIRA1 complementation vector pBIG4MRBrev–CoIRA1. We first amplified the upstream region of the *CoIRA1* gene, the middle region of the *CoIRA1* gene, and the downstream region of the *CoIRA1* gene by PCR using the primer pairs CoIRA1F3A–CoIRA1R4D, CoIRA1F5D–CoIRA1R5C, and CoIRA1F4C–CoIRA1R4D, respectively. Next, the pBIG4MRBrev–CoIRA1 vector was constructed with the amplified product, and the *A. tumefaciens* binary vector pBIG4MRBrev was constructed using the GeneART seamless cloning and assembly kit. This vector, which contains a bialaphos resistance gene, was used as the gene replacement plasmid.

### Construction of dominant active and negative *CoRAS1* vectors

To construct a dominant active form of the *CoRAS1* vector, we amplified a 1.4-kb fragment containing the upstream region of the *CoRAS1* gene, a 2.0-kb fragment containing the downstream region of the *CoRAS1* gene, and the linearized pBIG4MRBrev vector by PCR using the primer pairs CoRAS1F1A–CoRAS1^G17V^R1B, CoRAS1^G17V^F1A–CoRAS1R1B, and CoRAS1pBIF1A–R1B. Next, the pBIG4MRBrev–CoRAS1^G17V^ vector was constructed with the amplified product using the GeneART seamless cloning and assembly kit. To construct a dominant negative form of the *CoRAS1* vector, we amplified a 1.4-kb fragment containing the upstream region of the *CoRAS1* gene, a 2.0-kb fragment containing the downstream region of the *CoRAS1* gene, and the linearized pBIG4MRBrev vector by PCR using the primer pairs RAS1^S22N^F1A–RAS1R1B, RAS1F1A–RAS1^S22N^R1B, and CoRAS1pBIF1A–R1B, respectively. Next, the pBIG4MRBrev–CoRAS1^S22N^ vector was constructed with the amplified product using the GeneART seamless cloning and assembly kit.

### Construction of the *CoRAS2* gene replacement, complementation, dominant active or negative vectors

To replace the *CoRAS2* gene for a hygromycin-resistance gene, we constructed the *CoRAS2* gene-replacement vector pBIG4MRBrev–coras2. We first amplified the upstream region of the *CoRAS2* gene, the hygromycin-resistance gene, the downstream region of the *CoRAS2* gene, and the linearized pBIG4MRBrev vector by PCR using the primer pairs CoRAS2F1A–CoRAS2R2D, CoRAS2hphF1C–CoRAS2hphR1D, CoRAS2F2C–CoRAS2R1B, and CoRAS2pBIF1–R1, respectively. Next, the pBIG4MRBrev–coras2 vector was constructed with the amplified product using the GeneART seamless cloning and assembly kit. To perform a complementation assay for the *coras2* mutant, we constructed the *CoRAS2* complementation vector pBIG4MRBrev–CoRAS2. We amplified a 3.3-kb fragment containing the *CoRAS2* gene and the linearized pBIG4MRBrev vector by PCR using the primer pairs CoRAS2F1A–R1B and CoRAS2pBIF1A–R1B, respectively. Next, the pBIG4MRBrev–CoIRA1 vector was constructed with the amplified product using the GeneART seamless cloning and assembly kit.

To construct a dominant active form of the *CoRAS2* vector, we amplified a 1.4-kb fragment containing the upstream region of the Co*RAS2* gene, a 1.9-kb fragment containing the downstream region of the *CoRAS2* gene, and the linearized pBIG4MRBrev vector by PCR using the primer pairs CoRAS2F1A–CoRAS2^Q65L^R1B, CoRAS2^Q65L^F1A–CoRAS2R1B, and CoRAS2pBIF1A–R1B, respectively. Next, the pBIG4MRBrev–CoRAS2^Q65L^ vector was constructed with the amplified product using the GeneART seamless cloning and assembly kit. To construct a dominant negative form of the CoRas2 vector, we amplified a 1.2-kb fragment containing the upstream region of the *CoRAS2* gene, a 2.2-kb fragment containing the downstream region of the *CoRAS2* gene, and the linearized pBIG4MRBrev vector by PCR using the primer pairs CoRAS2F1A–CoRAS2^G19A^R1B, CoRAS2^G19A^F1A–CoRAS2R1B, and CoRAS2pBIF1A–R1B, respectively. Next, the pBIG4MRBrev–CoRAS2^G19A^ vector was constructed with the amplified product using the GeneART seamless cloning and assembly kit.

### Construction of the *CoIRA1*–VENUS vector

To construct the pBITEF–VENUS vector, we amplified a 0.9-kb fragment containing the TEF promoter and the upstream region of the *GFP* gene, a 0.6-kb fragment containing the downstream region of the *GFP* gene, and the linearized pBIG4MRBrev vector by PCR using the primer pairs TEFF1–VENUSR1, VENUSF1–glyGFPR1, and pBIG4VENUSF1–R1, respectively. Next, the pBIG4MRBrev–TEF–VENUS vector was constructed with the amplified product using the GeneART seamless cloning and assembly kit. To construct the CoIRA1–VENUS vector, we amplified the *VENUS* gene, the hygromycin-resistance gene, and the linearized pBIG4MRBrev–CoIRA1 vector by PCR using the primer pairs glyGFPF1–GFPR1, VENUShphF1–VENUSR1, and pBICoIRA1–VENUSF1–CoIRApBIcomR1–GFP, respectively. Next, the pBIG4MRBrev–CoIRA1–VENUS–hph vector was constructed with the amplified product using the GeneART seamless cloning and assembly kit.

### Construction of the *RFP* fused *CoRAS2* and the *RFP* fused *CoRAS2* ^Q65L^ vectors

To construct the *RFP* fused *CoRAS2* vector, we amplified the *RFP* gene and linearized pBIG4MRBrev–CoRAS2 vector by PCR using the primer pairs RFPF1–glyRFPR1 and CoRAS2pBIF1–R1, respectively. Next, the pBIG4MRBrev–RFP–CoRAS2 vector was constructed with the amplified product using the GeneART seamless cloning and assembly kit. To construct the *RFP–*fused *CoRAS2* ^Q65L^ vector, we amplified the *RFP* gene and linearized pBIG4MRBrev–CoRAS2^Q65L^ vector using the primer pairs RFPF1–glyRFPR1 and CoRAS2pBIF1–R1, respectively. Next, the pBIG4MRBrev–RFP–CoRAS2^Q65L^ vector was constructed with the amplified product using the GeneART seamless cloning and assembly kit.

### Construction of the VENUS–N (1–158 aa) fused CoIRA1 and VENUS–C (159–238 aa) fused CoRAS2 vectors for BiFC assays

To construct the *VENUS*-N fused *CoIRA1* vector, we amplified the *VENUS*–N fragment, the hygromycin-resistance gene, and the linearized pBIG4MRBrev–CoIRA1 vector by PCR using the primer pairs glyGFPF1–αGFPR1, VENUShphF1–VENUSR1, and pBICoIRA1–VENUSF1–CoIRApBIcomR1–GFP respectively. Next, the pBIG4MRBrev–CoIRA1–nVENUS–hph vector was constructed with the amplified product using the GeneART seamless cloning and assembly kit. To construct the *VENUS*–C fused *CoRAS2* vector, we amplified the *VENUS*–C fragment and linearized pBIG4MRBrev–CoRAS2 vector by PCR using the primer pair BGFPF2–glyGFPR1 and CoRAS2PBIcompF3–c–CoRAS2PBIcompR3, respectively. Next, the pBIG4MRBrev–cVENUS–CoRAS2 vector was constructed with the amplified product using the GeneART seamless cloning and assembly kit.

### Intracellular cAMP measurements

Mycelia were collected from three-day old PSY liquid cultures and frozen in liquid nitrogen. All samples were lyophilized for 24 h and weighed. For every 10 mg of mycelia, 200 µl of ice-cold 6% trichloroacetic acid was added. The precipitate was removed by centrifugation at 2000×g for 15 min at 4°C, the supernatant was transferred to a new tube, and the TCA was extracted four times with five volumes of water-saturated ether. The concentration of cAMP was determined using the cAMP Biotrak Enzyme immunoassay system (GE Health Life Science, UK) according to the manufacturer’s instructions.

### Western blot

The total protein was isolated from vegetative hyphae using a previously described method [23]. The protein separated on SDS–PAGE gels was transferred onto a PVDF membrane using an Xcell SureLock Mini-Cell. The phosphorylation activation of Maf1 and Cmk1 MAPK kinase was detected using a PhosphoPlus p44/42 MAP kinase antibody kit (Cell Signaling Technology). Alkaline Phosphatase-conjugated secondary antibody and light emission from the enzymatic dephosphorylation of the CDP-Star Detection Reagent (GE Healthcare, Tokyo, Japan) was detected using the Fujifilm LAS-1000 Plus Gel Documentation System (Fujifilm, Tokyo). Anti-actin antibodies (Wako, Japan) were used at a 1∶1000 dilution for Western blot analysis.

### Pathogenicity tests

An inoculation assay on cucumber cotyledons (*Cucumis sativus* L. “Suyo”) was performed as described by Tsuji et al. (1997) [24]. The conidia of *C. orbiculare* were obtained from seven-day old cultures, and drops of 10-µl conidial suspension (1×10^5^ conidia per ml) were added on the surface of cucumber cotyledons at different locations. To assess invasive growth ability, 10-µl drops of spore suspension were added on wounded sites that were created by scratching the leaf surface with a sterile toothpick. After inoculation, the cotyledons were incubated at 24°C for seven days.

### Light microscopy

For appressorium formation and penetration assays *in vitro*, conidia were harvested from seven-day old PDA cultures and suspended in distilled water. The conidial suspension, adjusted to 1×10^5^ conidia per ml, was placed on a multiwell glass slide (eight-well multi-test slide; ICN Biomedicals, Aurora, OH, U.S.A.) and incubated in humid boxes at 24°C. Germlings were observed by a Nikon ECLIPSE E600 microscope with differential interference contrast optics (Nikon, Tokyo, Japan). To observe the formation of infectious hyphae in cucumber leaves, the conidial suspension was inoculated on the abaxial surface of cucumber cotyledons and incubated at 24°C for three days. Then, the inoculation site was cut off and stained with 0.1% lactophenol-aniline blue. VENUS and RFP fluorescence was observed by a Carl ZEISS Axio Imager M2 microscope (Zeiss, Gottingen, Germany) with 470 and 595 nm of excitation wavelength, respectively.

## Results

### Identification of an *IRA1/2* homolog, *CoIRA1*, in *C. orbiculare*

*CoIRA1* was first identified as a mutant gene in the *Agrobacterium tumefaciens*-mediated transformation (AtMT) T-DNA mutant AA4510 of *C. orbiculare*, which shows an attenuated pathogenicity on cucumber leaves. DNA flanking regions, adjacent to the inserted plasmid, were isolated from the mutant AA4510 by thermal asymmetrical interlaced-polymerase chain reaction (TAIL-PCR) and the amplified products were subsequently sequenced. The TAIL-PCR result showed that the T-DNA fragment was inserted probable 10-bp downstream from the translational origin of the gene ENH81573, based on *C. orbiculare* genomic information [25]. This gene putatively encodes a 2255-amino acid protein with a predicted RAS GTPase-activating protein (RASGAP) domain (Figure S1A). We named this gene *CoIRA1.*

A blast search of the *CoIRA1* fungal genome homologs in non-redundant protein database of the National Center for Biotechnology Information indicated that the derived amino acid sequence from this gene is significantly similar to that of the *IRA1/2* homolog in *S. cerevisiae* and filamentous fungi, including *C. graminicola*, *Neurospora crassa,* and *M. oryzae* (Figure S1B).

### *CoIRA1* is required for infection-related morphogenesis

To analyze the function of *CoIRA1*, the disruption vector pBIG4MRBrevcoira1 was designed to replace the wild-type *CoIRA1* gene with the hygromycin phosphotransferase (*hph*) gene, by double crossover homologous recombination. Successful replacement of the targeted gene in the transformants was confirmed by genomic DNA blot analysis (Figure S2). A single 3.4-kb band was detected in the wild type, whereas a single 5.6-kb band was detected in *coira1* mutants, as expected from such a targeted gene replacement event (Figure S2). In ectopic transformants, the 3.4-kb band and several additional bands were detected, indicating ectopic insertion events.

**Figure 1:**
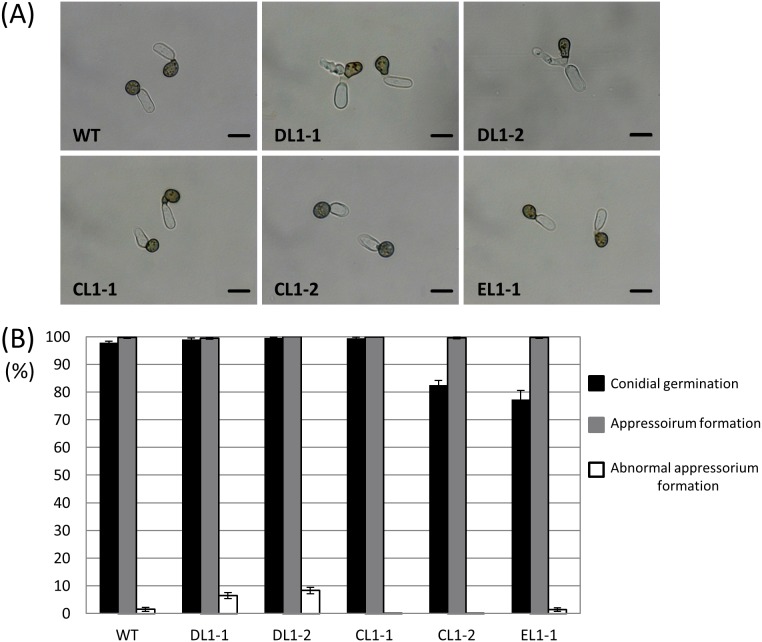
Appressorium formation in *coira1* mutants of *C. orbiculare* on glass slides. (A) Conidial suspensions of each strain in distilled water were incubated on multiwell glass slides at 24°C for 24 h. WT, the wild-type 104-T; DL1-1 and DL1-2, the *coira1* mutants; CL1-1, the *CoIRA1*-complemented transformant of DL1-1; CL1-2, the *CoIRA1*-complemented transformant of DL1-2; EL1-1, ectopic strain. Scale bar, 10 µm. (B) Percentages of conidial germination, appressorium formation, and abnormal appressorium formation in the *C. orbiculare* WT and *coira1* mutants on multiwell glass slides. Approximately 100 conidia of each strain were observed per well on multiwell slide glass. Three replicates were examined. Three independent experiments were conducted, and standard errors are shown. Black bar, conidial germination; gray bar, appressorium formation; white bar, abnormal appressorium formation.

**Figure 2:**
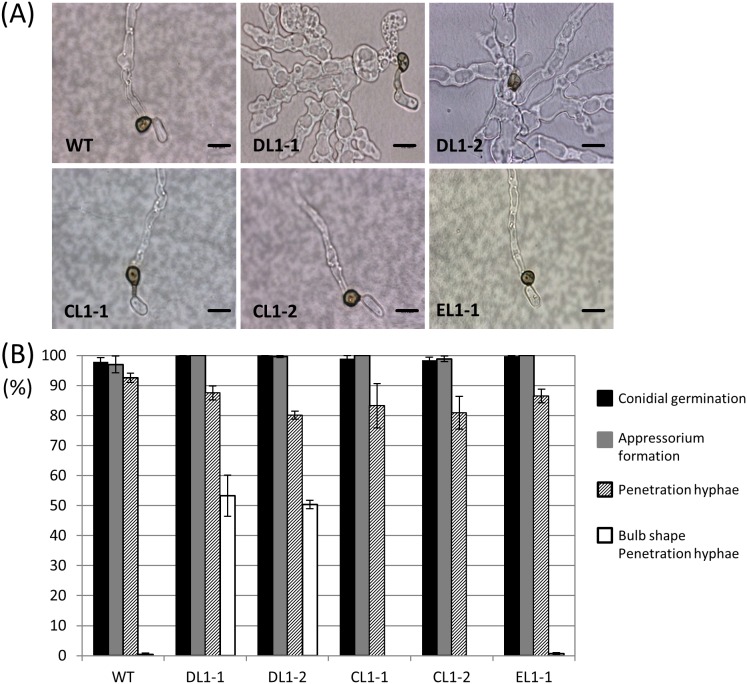
Penetration hyphae formation of the *coira1* mutants of *C. orbiculare* on cellulose membranes. Conidial suspensions of each strain in distilled water were incubated on cellulose membranes at 24°C for 48 h. WT, the wild-type 104-T; DL1-1 and DL1-2, the *coira1* mutant; CL1-1, the *CoIRA1*-complemented transformant of DL1-1; CL1-2, the *CoIRA1*-complemented transformant of DL1-2; EL1-1, the ectopic strain. Scale bar, 10 µm. (B) Percentages of conidial germination, appressorium formation, penetration hyphae formation, and bulb-shaped penetration-hyphae formation of *C. orbiculare* WT and *coira1* mutants on cellulose membranes. Approximately 200 conidia of each strain were observed on cellulose membranes. Three replicates were examined. Three independent experiments were conducted, and standard errors are shown. Black bar, conidial germination; gray bar, appressorium formation; slash bar, penetration hyphae; white bar, bulb-shape penetration formation.

The hyphal growth of the *coira1* mutant was similar to that of the wild-type, but the *coira1* mutant formed a rather dark colony compared with the wild-type (Figure S3). The *coira1* mutant also showed reduced conidiation compared with the wild-type. To investigate infection-related morphogenesis in the *coira1* mutant, we observed conidial germination and appressorium development on a glass slide, and infection hyphae development on cellulose membranes. In the wild-type, *coira1* mutant, ectopic transformants, and *CoIRA1* reintroduced transformants, approximately 80% of the conidia germinated and formed darkly melanized appressoria after 24 h, but the frequency of abnormal appressorium formation in the *coira1* mutant was higher compared with the wild-type, as observed on glass slides (Figures 1A and B). The appressorium turgor pressure is required to penetrate the plant surface mechanically during infection [26], therefore, we evaluated the appressorium turgor in the *coira1* mutant using a cytorrhysis assay [27]. The proportion of the collapsed appressoria at each glycerol concentration in the *coira1* mutant was similar to that of the wild-type (Figure S4), indicating that CoIra1 is not involved in the generation of the appressorium turgor pressure. The wild-type, ectopic transformants, and *CoIRA1* reintroduced transformants formed normal infection-hyphae that penetrated the cellulose membranes with high frequency. The *coira1* mutant effectively formed infection hyphae as did the wild-type. Interestingly, the *coira1* mutant hyphae had a bulbous shape, which was quite different from those of the wild-type (Figures 2A and B). These results indicated that *CoIRA1* is involved in the progression of normal morphogenesis during infection.

### *CoIRA1* is involved in the pathogenicity of the host plant

**Figure 3:**
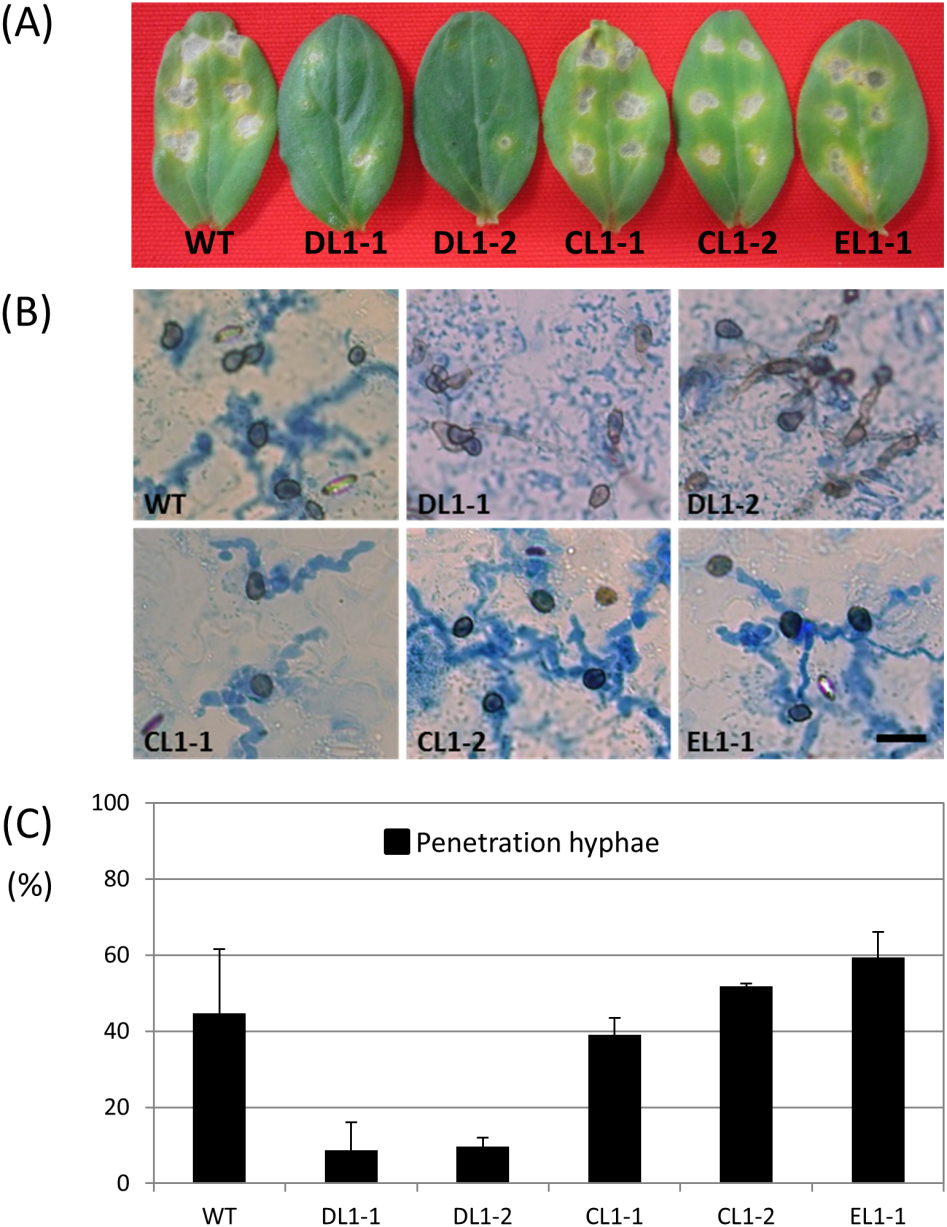
Pathogenicity assay and penetration ability of *coira1* mutants of *C. orbiculare* on the cucumber cotyledons. (A) Conidial suspensions of each strain (10 µl) placed on detached cucumber cotyledons and leaves incubated at 24°C for three days. The figure shows the leaves after incubation with the following strains: WT, the wild-type 104-T; DL1-1 and DL1-2, the *coira1* mutant; CL1-1, the *CoIRA1*-complemented transformant of DL1-1; CL1-2, the *CoIRA1*-complemented transformant of DL1-2; EL1-1, the ectopic strain. (B) Penetration hyphae development of each strain on the cucumber cotyledons. Conidial suspensions (10 µl) were applied to the abaxial surface of the cucumber cotyledons and incubated at 24°C for 72 h. Scale bar, 20 µm. (C) Percentage of penetration hyphae of the *coira1* mutants on the abaxial surface of cucumber cotyledons. Approximately 100 appressorium were observed per incubated site. Three replicates were examined. Three independent experiments were conducted, and standard errors are shown. Black bar, penetration hyphae. Scale bar, 20 µm.

To investigate whether the pathogenicity of *coira1* mutants is attenuated, conidial suspensions were inoculated onto cucumber cotyledons. The pathogenicity of the *coira1* mutant was found to be attenuated during the infection of the cucumber plants compared with that of the wild-type (Figure 3A). Moreover, when conidial suspensions were applied directly on wounded sites, the *coira1* mutant caused the formation of smaller lesions compared with the wild-type (Figure S5). Microscopic observations of the infection process of the *coira1* mutant showed that it formed normal darkly melanized appressoria and infection hyphae, however, its frequency of infection hyphae formation was lower than that of the wild-type (Figures 3B and C). These data indicated that during the infection of the cucumber, the *coira1* mutants had a defective infection-related morphogenesis. In *C. orbiculare*, we showed that the *ssd1* mutant that are defective in proper fungal cell walls constitution failed to penetrate into host epidermal cells due to the increased defence reaction of the host plant with rapidly induced callose deposition at at the attempted penetration site from appressoria [28]. Therefore, to investigate whether the observed low frequency of the infection hyphae formation in the *coira1* mutant was induced by a host defense mechanism, we monitored the callose deposition under the appressorium of the *coira1* mutant on the cucumber cotyledons and found that the extent and frequency of the deposition was similar to that of the wild-type (Figure S6A). Moreover, the *coira1* mutant caused the formation of smaller lesions than the wild-type on heat-shocked cucumber cotyledons (Figure S6B), which impaired the host defense responses, indicating that the induction of plant defense responses is not involved in the attenuated pathogenicity observed for *coira1* mutants.

### *CoIRA1* is involved in cAMP signaling

**Figure 4:**
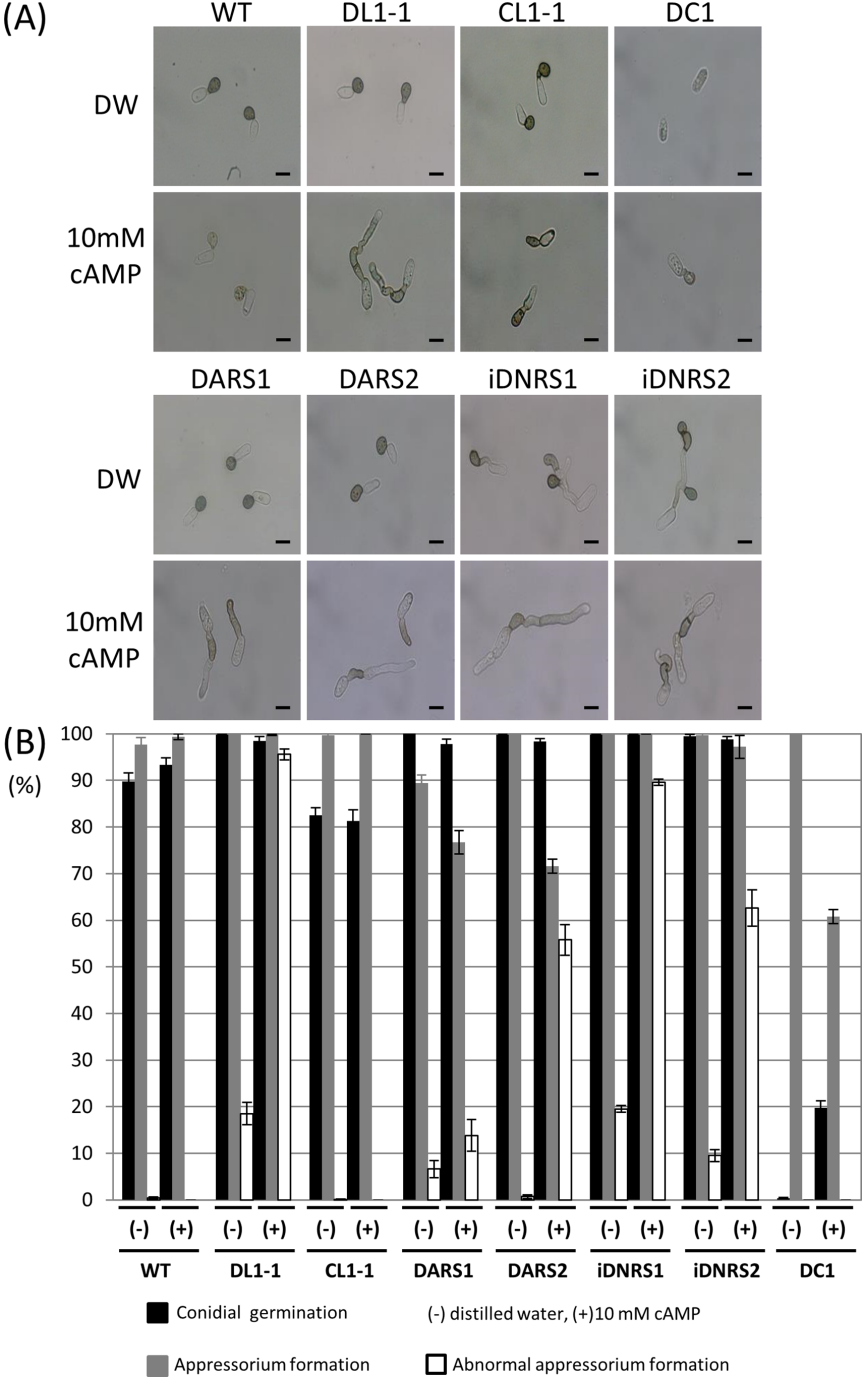
Appressorium formation by *coira1* mutants on glass slide in the presence of 10-mM cAMP. (A) Conidial suspensions of each strain in distilled water or 10-mM cAMP were incubated on the multiwell glass slide at 24°C for 24 h. WT, the wild-type 104-T; DL1-1, the *coira1* mutant; CL1-1, the *CoIRA1*-complemented transformant of DL1-1; DC1, the *cac1* mutant; DARS1, WT transformed with a dominant active form *CoRAS1*; DARS2, WT transformed with a dominant active form *CoRAS2*; iDNRS1, DL1-1 transformed with a dominant negative form *CoRAS1*; iDNRS2, DL1-1 transformed with a dominant negative form *CoRAS2*. Scale bar, 10 µm. (B) Percentages of conidial germination, appressorium formation, and abnormal appressorium formation of *C. orbiculare* on multiwell glass slides in the presence of 10-mM cAMP. Approximately 100 conidia of each strain were observed on multiwell glass slides. Three replicate experiments were examined. Three independent experiments were conducted, and standard errors are shown. Black bar, conidial germination; gray bar, appressorium formation that includes normal appressorium and abnormal appressorium; white bar, abnormal appressorium formation. (–) distilled water, (+) 10-mM cAMP.

In *S. cerevisiae*, Ira1/2 inactivates Ras1/2, which in turn activates adenylate cyclase, leading to the synthesis of cAMP from ATP [29]. We therefore examined whether the appressorium morphogenesis of the *coira1* mutants is affected by exogenous cAMP signals. In the presence of 10 mM cAMP, the frequency of abnormal appressorium formation observed in the *coira1* mutant was higher compared with that of the wild-type (Figures 4A and B). This data suggested that CoIra1 is involved in cAMP signaling pathway during the process of the appressorium formation. We identified two Ras genes and named *CoRAS1* (ENH84705) and *CoRAS2* (ENH80898). The comparative amino acid sequence analysis of Ras proteins showed that *CoRAS1* was 90% identical to *M. oryzae MoRAS1* and *CoRAS2* was 80% identical to *M. oryzae MoRAS2*. To better understand relationship between CoIra1 and Ras proteins, we set up a hypothesis that the wild-type expressing a dominant active form *CoRAS1* and *CoRAS2* increases abnormal appressorium formation compared with the wild-type in the presence of excess cAMP, while the *coira1* mutant expressing a dominant active form *CoRAS*1 and *CoRAS2* decrease abnormal appressorium compared with the *coira1* mutant in the presence of excess cAMP. Therefore, we generated these transformants. The Ras protein shows a high degree of amino acid conservation. On the basis of the human Ras gene mutation information, we constructed the dominant active forms CoRas1 (*RAS1* ^G17V^) and CoRas2 (*RAS2* ^Q65L^) alleles by replacing Gln-65 with Leu and Gly-17 with Val, respectively. Moreover, based on *C. trifolii* and *Candida albicans* Ras gene mutation information, we constructed the dominant negative forms CoRas1 (*RAS1* ^S22N^) and CoRas2 (*RAS2* ^G19A^) alleles by replacing Ser-22 with Asn and Gly-19 with Ala, respectively [6], [30]–[32]. Next, we generated the dominant active and negative forms of Co*RAS1* and Co*RAS2* of *C. orbiculare*. DARS1 (Dominant Active form *CoRAS1* ^G17V^) is the wild-type transformant, expressing the dominant active form *CoRAS1* ^G17V^ and DARS2 (Dominant Active form *CoRAS2* ^Q65L^) is the wild-type transformant, expressing the dominant active form *CoRAS2^Q65L^*. On the other hand, iDNRS1 (the *coira1*/Dominant Negative form *CoRAS1* ^S22N^) is the *coira1* transformant, expressing the dominant negative form *CoRAS1* ^S22N^ and iDNRS2 (the *coira1*/Dominant Negative form *CoRAS2* ^G19A^) is the *coira1* transformant, expressing the dominant negative form *CoRAS2* ^G19A^.

**Figure 5:**
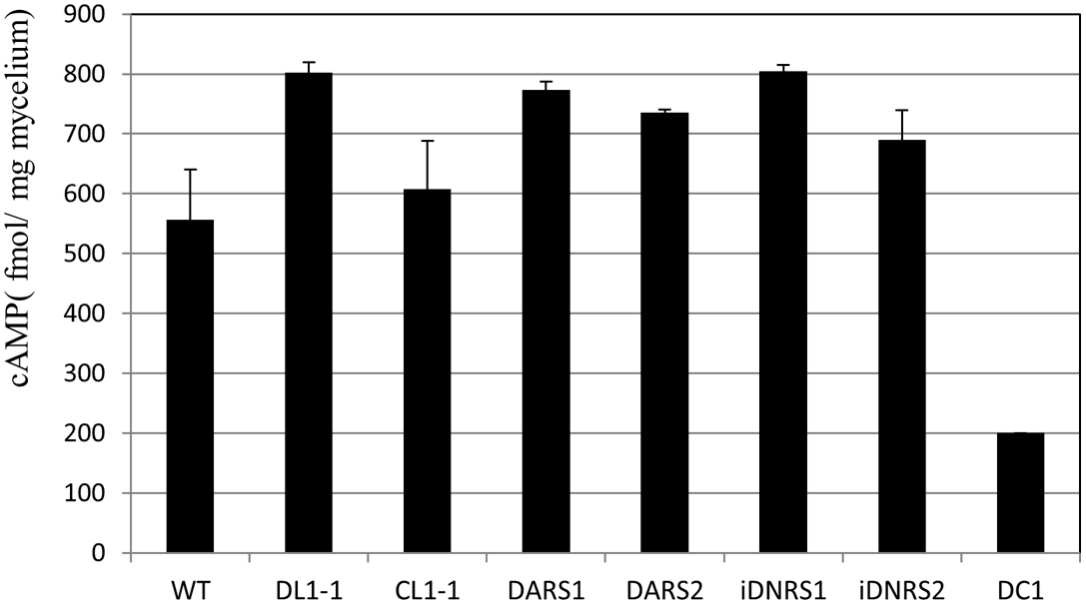
Intracellular cAMP levels in the *coira1* mutant. Intracellular cAMP levels were measured in tissue collection from three-day old liquid cultures of each strain. WT, the wild-type 104-T; DL1-1, the *coira1* mutant; CL1-1, the *CoIRA1*-complemented transformant of DL1-1; DARS1, WT transformed with a dominant active form *CoRAS1*; DARS2, WT transformed with a dominant active form *CoRAS2*; iDNRS1, DI1-1 transformed with a dominant negative form *CoRAS1*; iDNRS2, DI1-1 transformed with a dominant negative form *CoRAS2*; DC1, the *cac1* mutant. Three independent experiments were conducted, and standard errors are shown.

DARS1 and DARS2 showed normal appressorium formation on the glass slides in distilled water (Figure 4) and normal penetration hyphae similar to the wild-type on the cellulose membranes (Figure S7), however, DARS2 caused the formation of smaller lesions compared with the wild-type and DARS1 on the cucumber cotyledons (Figure S8). In the presence of 10 mM cAMP, DARS2 formed abnormal appressoria, similar to those observed for the *coira1* mutants. In contrast, iDNRS2 suppressed the abnormal appressorium formation compared with that of the *coira1* mutants (Figure 4). These data indicated that CoIra1 could control the cAMP signaling pathway through CoRas2 during the process of appressorium formation. We also checked the intracellular cAMP accumulation in the *coira1* mutant and the dominant active and negative *CoRAS1* and *CoRAS2* introduced transformants in vegetative hyphae whether the cAMP signaling pathway was regulated by those genes. The *coira1* mutants accumulated higher levels of cAMP compared with the wild-type (Figure 5). Moreover, intracellular cAMP levels in the DARS1 and DARS2 mutants were similar to those of the *coira1* mutant. These data indicated that intracellular cAMP levels in the vegetative hyphae were controlled by CoIra1, CoRas1, and CoRas2.

### *CoRAS2* is involved in conidial germination and pathogenicity

CoIra1 regulates intracellular cAMP levels through Ras proteins. Therefore, to analyze the functional roles of *CoRAS1* and *CoRAS2*, we aimed to generate *coras1* and *coras2* mutants by AtMT. Whereas we successfully developed *coras2* mutants, the generation of *coras1* mutants was challenging, indicating that *CoRAS1* could be an essential gene in *C. orbiculare*. To investigate whether *CoRAS2* is involved in infection-related morphogenesis, we observed conidial germination and appressorium formation on glass slides. Strikingly, conidial germination was not observed on glass slides for *coras2* mutants, indicating that *CoRAS2* is involved in conidial germination (Figure S9). To investigate whether the pathogenicity of the *coras2* mutant was attenuated during the infection of the cucumber cotyledons, conidial suspensions were inoculated onto the cucumber cotyledons. The *coras2* mutant was defective in pathogenesis to the cucumber cotyledons, forming small speck lesion (Figure S10). Microscopic observation revealed that most conidia in the *coras2* mutant were defective in conidial germination as *in vitro* condition, indicating that the attenuated pathogenicity of this mutant was due to a defect in conidial germination and that small speck lesion was apparently caused by small numbers of appressorium forming conidia.

### CoRas2 is an upstream regulator of the MAPK signaling pathway

**Figure 6:**
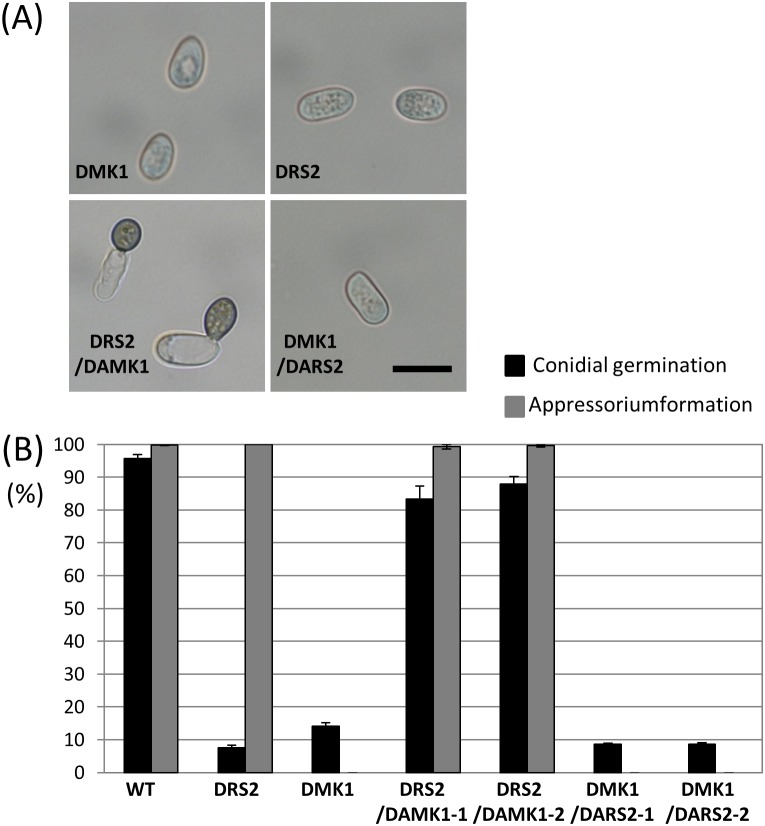
Appressorium formation assay of DRS2/DAMK1 and DMK1/DARS2 on the glass slides. (A) Conidial suspensions of each strain in distilled water incubated on multiwell glass slides at 24°C for 24 h. DRS2, the *coras2* mutant; DMK1, the *comekk1* mutant; DRS2/DAMK1, DRS2 transformed with a dominant active form *CoMEKK1*; DMK1/DARS2, DMK1 transformed with a dominant active form *CoRAS2*. Scale bar, 10 µm. (B) Percentages of conidial germination and appressorium formation in *C. orbiculare* on multiwell glass slides. Approximately 100 conidia of each strain were observed per well on the multiwell slide glass. Three replicates were examined. Three independent experiments were conducted, and standard errors are shown. Black bar, conidial germination; gray bar, appressorium formation.

From previous reports, the MAPK CoMekk1–Cmk1 signaling pathway has been shown to play pivotal roles in conidial germination and appressorium formation in several *Colletotrichum* speciese [2], [33]. In *C. orbiculare*, the Ste11 homolog *CoMEKK1* encodes a Ras-association domain. Thus, to analyze whether CoRas2 is an upstream regulator of CoMekk1, we generated a *comekk1* transformant DMK1/DARS2 expressing a dominant active form of the *CoRAS2* allele. In addition, we generated the *coras2* mutant DRS2/DAMK1 expressing a dominant active form of the *CoMEKK1* allele. A normal conidial germination and appressorium formation was observed on the glass slide for the DRS2/DAMK1 mutant (Figure 6). On the other hand, the defective conidial germination of the *coras2* mutant remained in the DMK1/DARS2 mutant, showing a similar frequency of conidial germination as that in the *coras2* mutant. These data suggested that *CoRAS2* is an upstream regulator of the MAPK CoMekk1–Cmk1 signaling pathway.

**Figure 7:**
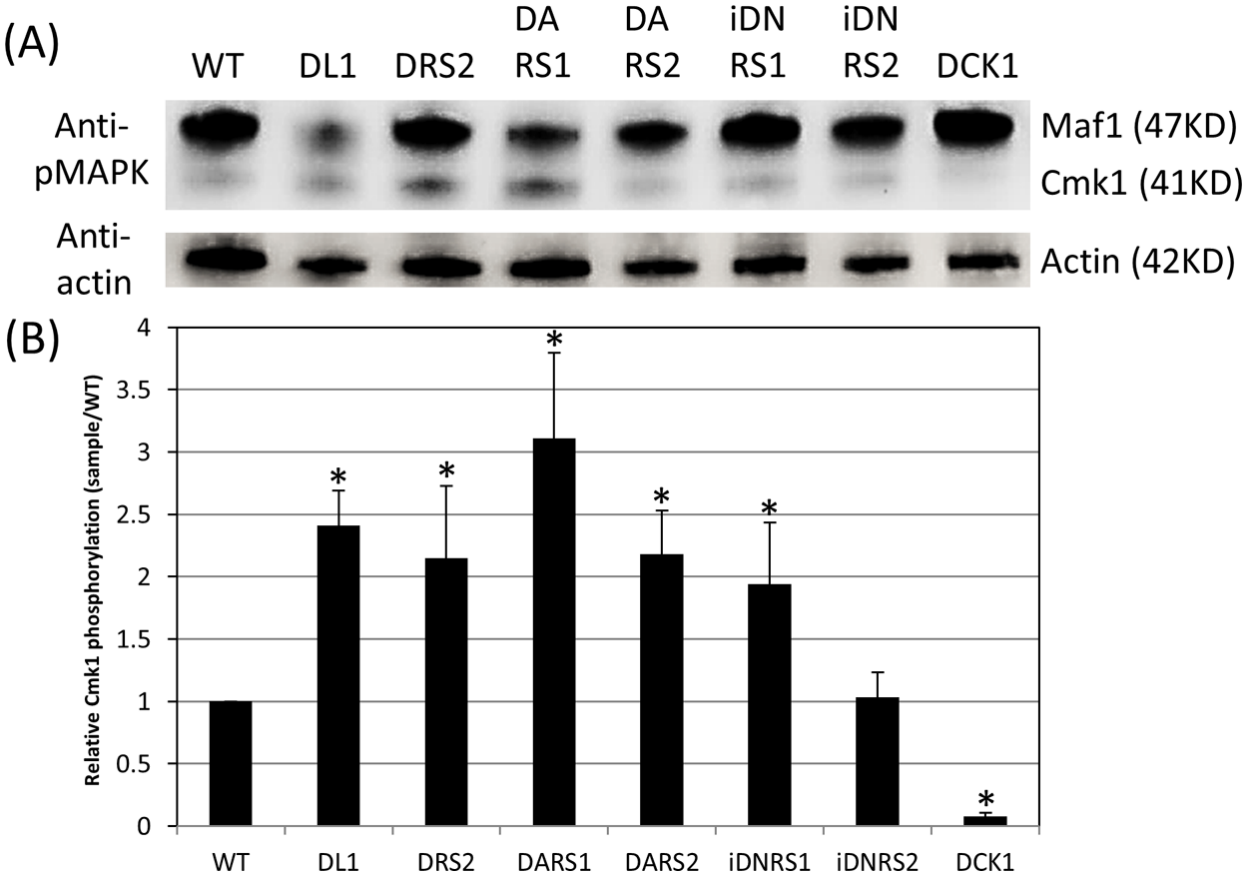
The phosphorylation of MAPK Cmk1 in the *coira1* mutant. (A) The total protein isolated from mycelia of each strain. WT; the wild-type, DL1; the *coira1* mutant, DRS2; the *coras2* mutant, DARS1, WT transformed with a dominant active form *CoRAS1*, DARS2; WT transformed with a dominant active form *CoRAS2*, iDNRS1; the *coira1* mutant transformed with a dominant negative form *CoRAS1*, iDNRS2; the *coira1* mutant transformed with a dominant negative form *CoRAS2,* DCK1; the *cmk1* mutant The anti-phospho p44/42 MAPK antibody detected a 41-KD Cmk1 and 47-KD Maf1. The anti-actin antibody detected a 42-KD actin. (B) Relative activity of MAPK Cmk1 phosphorylation of each mutant was calculated by comparison of signal intensity with that of the wild-type, normalized by actin signal. The quantitative analysis of phosphorylated Cmk1 was performed by four replicated experiments. Asterisk represents significant differences between the wild type and each mutant. (Student’s *t* test: *indicate *P*<0.05).

The MAP kinase Cmk1 is activated through threonine/tyrosine phosphorylation catalyzed by MAPKK, which at the same time is activated through serine phosphorylation catalyzed by CoMekk1. To determine whether the phosphorylation of Cmk1 in the *coira1* and *coras2* mutants was affected in the vegetative hyphae, we investigated the Thr–Gln–Tyr residue phosphorylation of Cmk1 using an anti-TpEY specific antibody. The phosphorylation levels of Cmk1 in the *coira1* mutant and DARS2 were higher compared with those in the wild-type, whereas phosphorylation levels of Cmk1 in iDNRS2 were lower compared with those in the *coira1* mutant, which was similar to those of the wild-type (Figure 7). These data indicated that CoIra1 negatively regulates the phosphorylation of Cmk1 through CoRas2. Interestingly, the phosphorylation level of Cmk1 in DARS1 was higher compared with that in the wild-type, indicating that CoRas1 positively regulates the phosphorylation of MAPK Cmk1.

### CoRas2 localization in the *coira1* mutant was similar to the active CoRas2 localization pattern in vegetative hyphae

**Figure 8:**
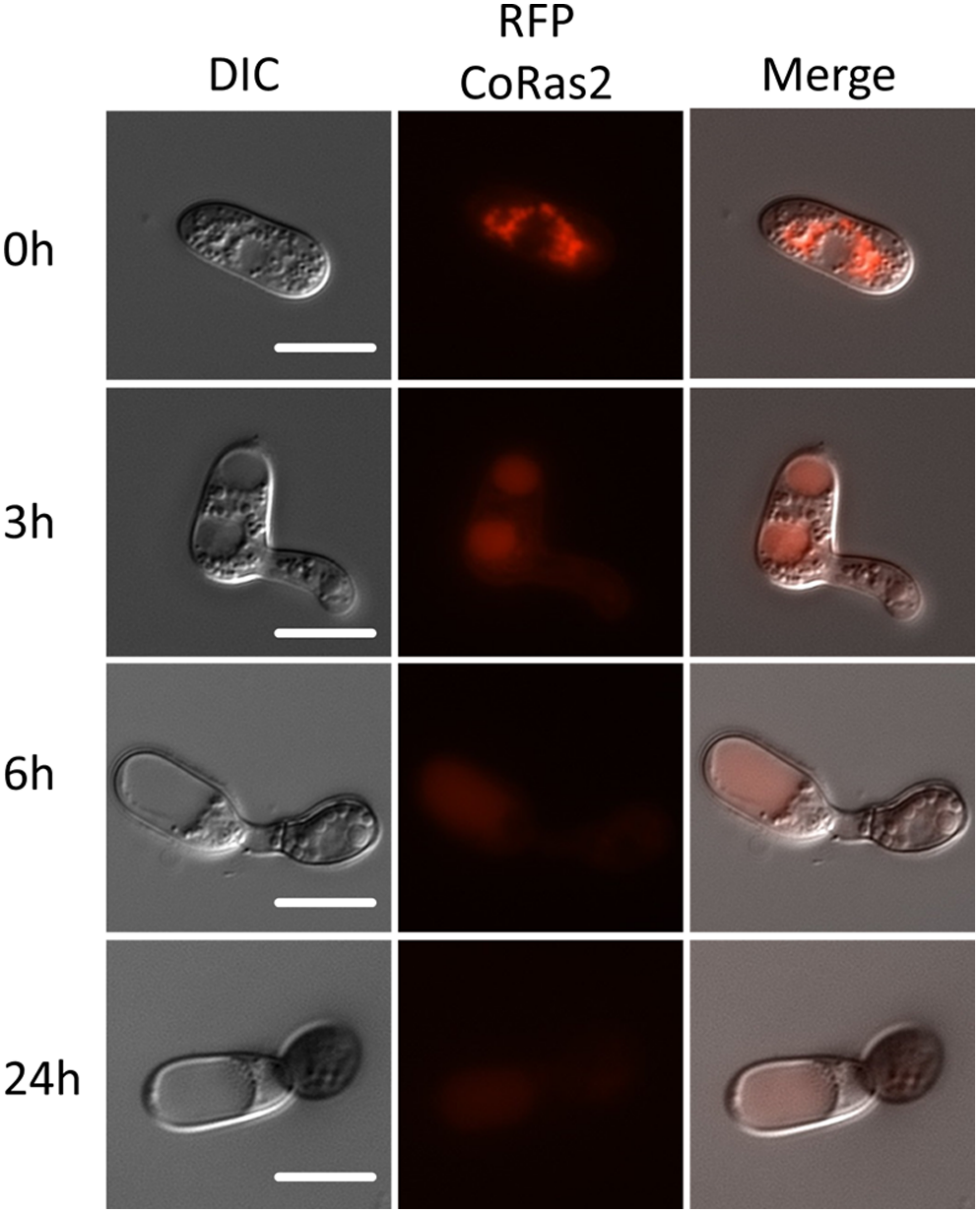
Localization of a functional RFP–CoRas2 fusion protein in *C. orbiculare* during conidial germination and appressorium formation. Conidial suspensions of the DRS2/RFP–RS2strain in distilled water were incubated in glass slides at 24°C for 0 h, 3 h, 6 h, and 24 h. After incubation, RFP fluorescence was observed by fluorescence microscopy. DRS2/RFP–RS2, the *coras2* mutant expressing *RFP–CoRAS2*. Scale bar, 10 µm.

**Figure 9:**
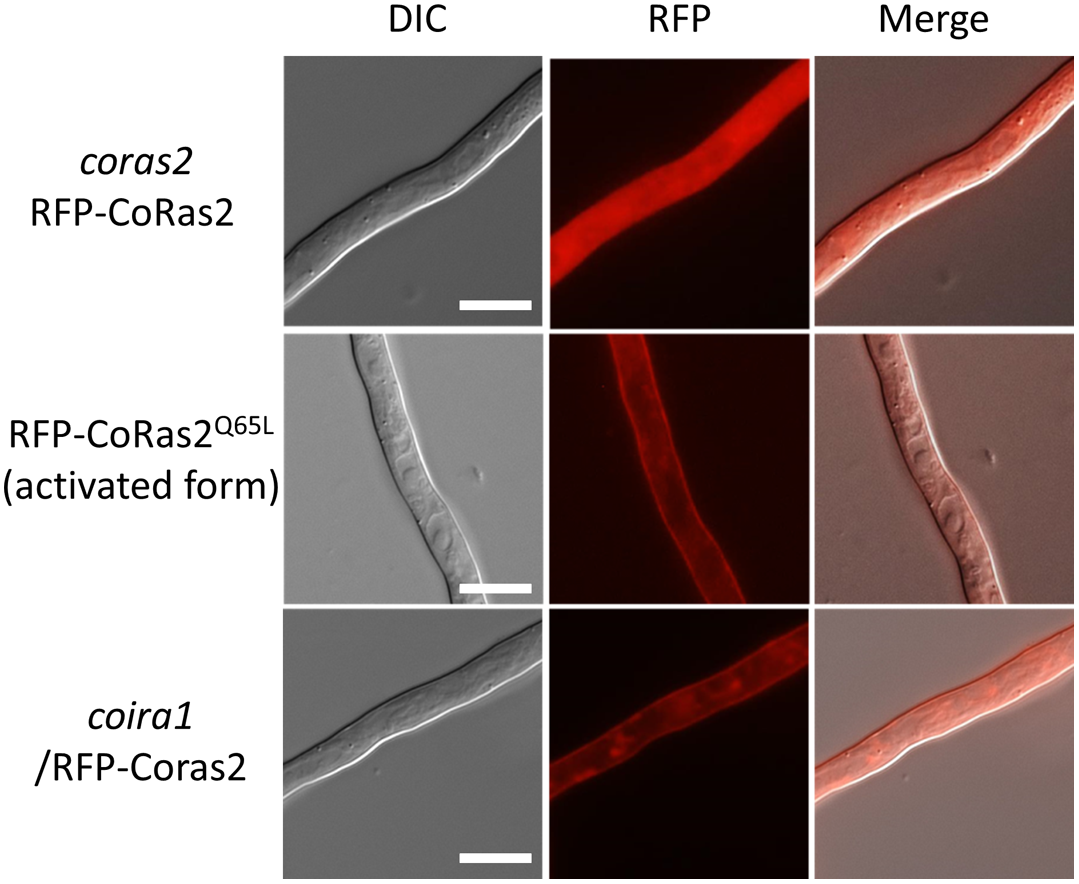
CoRas2 localization was regulated by CoIra1 in vegetative hyphae. Conidia harvested from each strain were observed on glass slides by fluorescent microscopy. *coras2*/RFP–*CoRAS2*, the *coras2* mutant expressing the *RFP–CoRAS2*; RFP–CoRas2^Q65L^, the wild-type strain expressing *RFP–CoRAS2* ^Q65L^; and *coira1*/RFP–CoRas2, the *coira1* mutant expressing *RFP–CoRAS2*. Scale bar, 10 µm.

**Figure 10:**
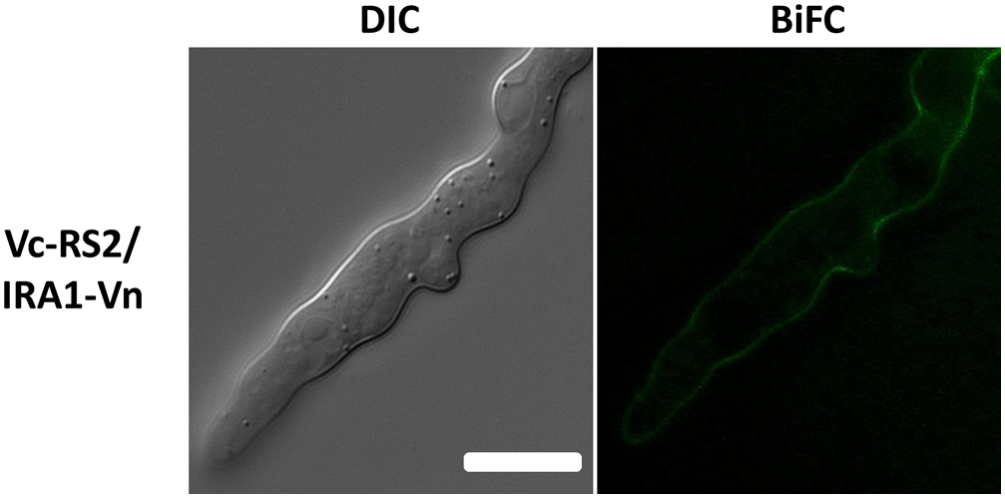
BiFC assays for CoIra1 and CoRas2 interactions in vegetative hyphae. Conidial suspensions of Vc-CoRas2/CoIra1-n transformant in liquid PSY medium were incubated at 28°C for 24 h and BiFC fluorescence was observed by fluorescent microscopy in vegetative hyphae. Vc–CoRas2/CoIra1–Vn; the wild-type strain expressing Vc–*CoRAS2* and Vn–*CoIRA1*. Scale bar, 10 µm.

To analyze the cellular CoRas2 localization, a red fluorescent protein (RFP) gene was fused to the C-terminus of the *CoRAS2* gene. In *S. cerevisiae* and *C. albicans*, the Ras protein conserves a CAAX motif (C, cysteine; A, aliphatic amino-acids; X; methionine or serine), which is important for post-translational modification, including farnesylation and palmitoylation, to ensure specific membrane localization and function [29], [34]. We assumed that the *RFP gene* fused C-terminus of *CoRAS2* gene may block the localization and function of CoRas2. Thus, the *RFP* gene fused to the N-terminus of *CoRAS2* gene was constructed, and the *RFP*–*CoRAS2* gene controlled under the *CoRAS2* native promoter was transformed into the *coras2* mutant (DRS2/RFP–RS2) and the wild-type (WT/RFP–RS2). Both DRS2/RFP–RS2 and WT/RFP–RS2 retained normal infection-related morphogenesis and pathogenicity. The signals of RFP–CoRas2 were detected within vesicle-like structures in conidia (Figure 8). During the process of premature appressoria formation, signals of the RFP–CoRas2 were localized mainly in subcellular compartments resembling vacuole-like structures in conidia, but these signals were not detected in matured appressoria. In vegetative hyphae, RFP–CoRas2 proteins showed no specific localization and their signals were uniformly distributed throughout the entire cell (Figure 8). To analyze the cellular localization of the active form of CoRas2, the *RFP* gene was fused to the N-terminus of the dominant active form of the *CoRAS2* gene. The *RFP*–DA*CoRAS2* gene controlled under the native *CoRAS2* promoter was transformed into the wild-type (WT/RFP–DARS2). During the process of appressoria formation, the signal pattern of the RFP–DACoRas2 was similar to that of the RFP-CoRas2 (Figure S11), however, the signals of the RFP–DACoRas2 in vegetative hyphae were detected predominantly in the plasma membrane, unlike those of native CoRas2. To analyze whether CoIra1 negatively regulates CoRas2, we generated an iRFP–RS2 transformant expressing *RFP*–*CoRAS2* in the *coira1* mutant and observed the cellular localization of CoRas2 in this mutant. During appressoria formation, the signals of RFP-CoRas2 in the *coira1* mutant were detected as vesicle like structures as well as RFP-CoRas2 and RFP-DACoRas2. In vegetative hyphae, the signals of RFP-CoRas2 in the *coira1* mutant were detected at the plasma membrane as well as RFP-DACoRas2 localization (Figure 9). Engagement of CoIra1 in the regulation of CoRas2 during appressorium formation was not directly suggested by these data from the viewpoint of the cellular localization of Ras protein, whereas it was suggested that CoIra1 functions as a negative regulator for CoRas2 in vegetative hyphae. Furthermore, to analyze whether CoIra1 regulates the CoRas2 in the plasma membrane in vegetative hyphae, we generated the Vc–RS2/IRA–Vn transformant, expressing the C-terminal domain (159–238) *VENUS* that was fused with *CoRAS2* and the N-terminal domain (1–158) of *VENUS* fused with *CoIRA1* in the wild-type for bimolecular fluorescence complementation (BiFC) assays [35]. BiFC fluorescence was detected at the plasma membrane in vegetative hyphae. This result indicated that CoIra1 regulates CoRas2 at the plasma membrane in vegetative hyphae (Figure 10).

### CoIra1 colocalizes with CoRas2 in pregerminated conidia and appressoria that initiate the differentiation of infection hyphae

**Figure 11:**
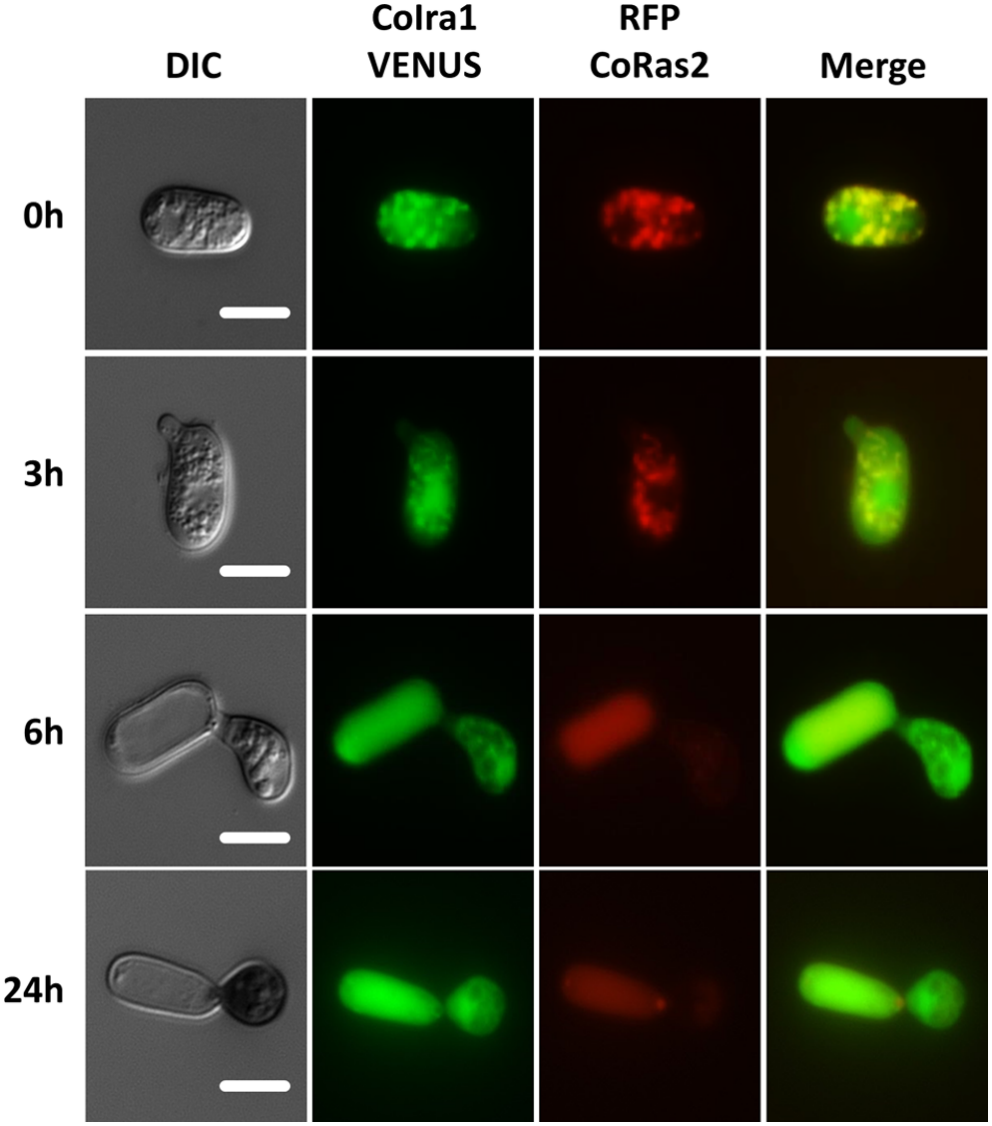
Assay for colocalization of CoIra1 and CoRas2. Conidial suspensions of the RFP–RS2/IRA1–VENUS strain were incubated on glass slides at 24°C for 0 h, 3 h, 6 h, and 24 h and observed by fluorescent microscopy. RFP–RS2/IRA1–VENUS, the wild-type strain expressing *CoIRA1*–VENUS and *RFP–CoRAS2*. Scale bar, 10 µm.

**Figure 12:**
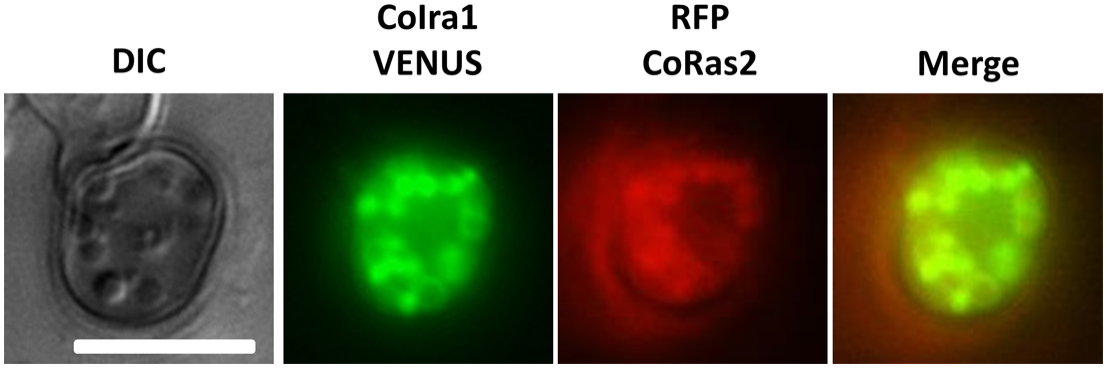
Assay for the colocalization of CoIra1 and CoRas2 in appressoria at 48 h after inoculation on cucumber leaves. Conidial suspensions of RFP–RS2/IRA1-strain were incubated in cucumber leaves at 24°C for 48 h and observed under fluorescent microscopy. RFP–RS2/IRA1–VENUS, the wild-type strain expressing *CoIRA1*–VENUS and *RFP–CoRAS2*. Scale bar, 10 µm.

To elucidate the cellular localization of CoIra1, the *VENUS* fluorescence gene was fused to the C-terminal of the *CoIRA1* gene [36]. *VENUS* fused with *CoIRA1*, expressing under a native *CoIRA1* promoter was transformed into the wild-type (IRA1–VENUS). CoIra1–VENUS was detected in a vesicle-like structure of conidia (Figure 11). In the germinated conidia, CoIra1–VENUS was detected at the tip of the germ tubes and distributed uniformly in the conidial cells. In developing appressoria, CoIra1–VENUS did not show any specific localization, and uniform signals were distributed throughout the appressoria except the presumable lipid bodies, similar to those on cucumber leaves. In developing infection hyphae, CoIra1–VENUS showed a uniform distribution either in the initial or late infection hyphae in cucumber cotyledons (Figure S12). Then, to elucidate the intracellular colocalization of CoIra1 and CoRas2, we generated the RFP–RS2/IRA1–VENUS and RFP–DARS2/IRA1–VENUS transformants by introducing the *RFP* fused with *CoRAS2* and *VENUS* fused with *CoIRA1* genes into the wild-type, and the *RFP* fused with a dominant active form *CoRAS2* and *VENUS* fused with *CoIRA1* genes into the wild-type, respectively. RFP–CoRas2 and CoIra1–VENUS colocalized at vesicle-like structures in conidia and germinated conidia (Figure 11), however, the RFP–CoRas2 and CoIra1–VENUS did not show specific colocalization in developing appressoria, similar to RFP–DARS2/IRA1–VENUS (Figure S11). Surprisingly, after 48 h of conidial incubation on glass slides, RFP–CoRas2 and CoIra1–VENUS were colocalized in a vesicle-like structures in appressoria (Figure 12). Conclusively, CoIra1 colocalizes with CoRas2 in pregerminated conidia and in appressoria that initiate differentiation of infection hyphae.

### CoIra1 regulates the assembly of actin at the appressorium pore

**Figure 13:**
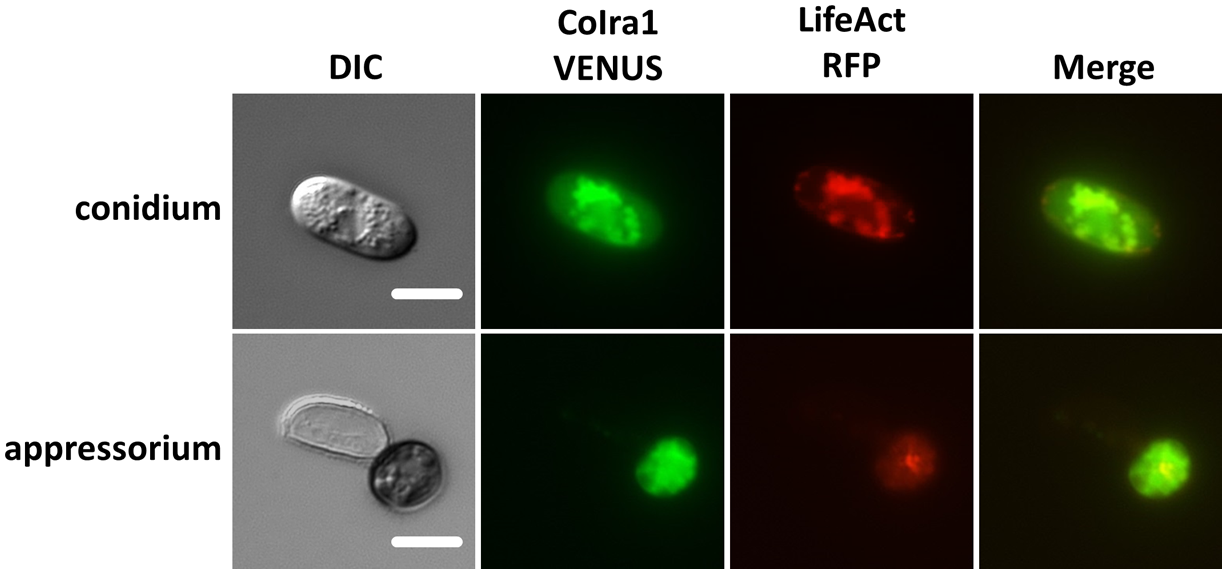
Assay for colocalization of CoIra1 and F-actin. Conidial suspensions of the LA/IRA1–VENUS strain were incubated on glass slides at 24°C for 0 h and 24 h and observed under fluorescent microscopy. LA/IRA1–VENUS, the wild-type strain expressing *CoIRA1*–VENUS and Lifeact–*RFP.* Scale bar, 10 µm.

**Figure 14:**
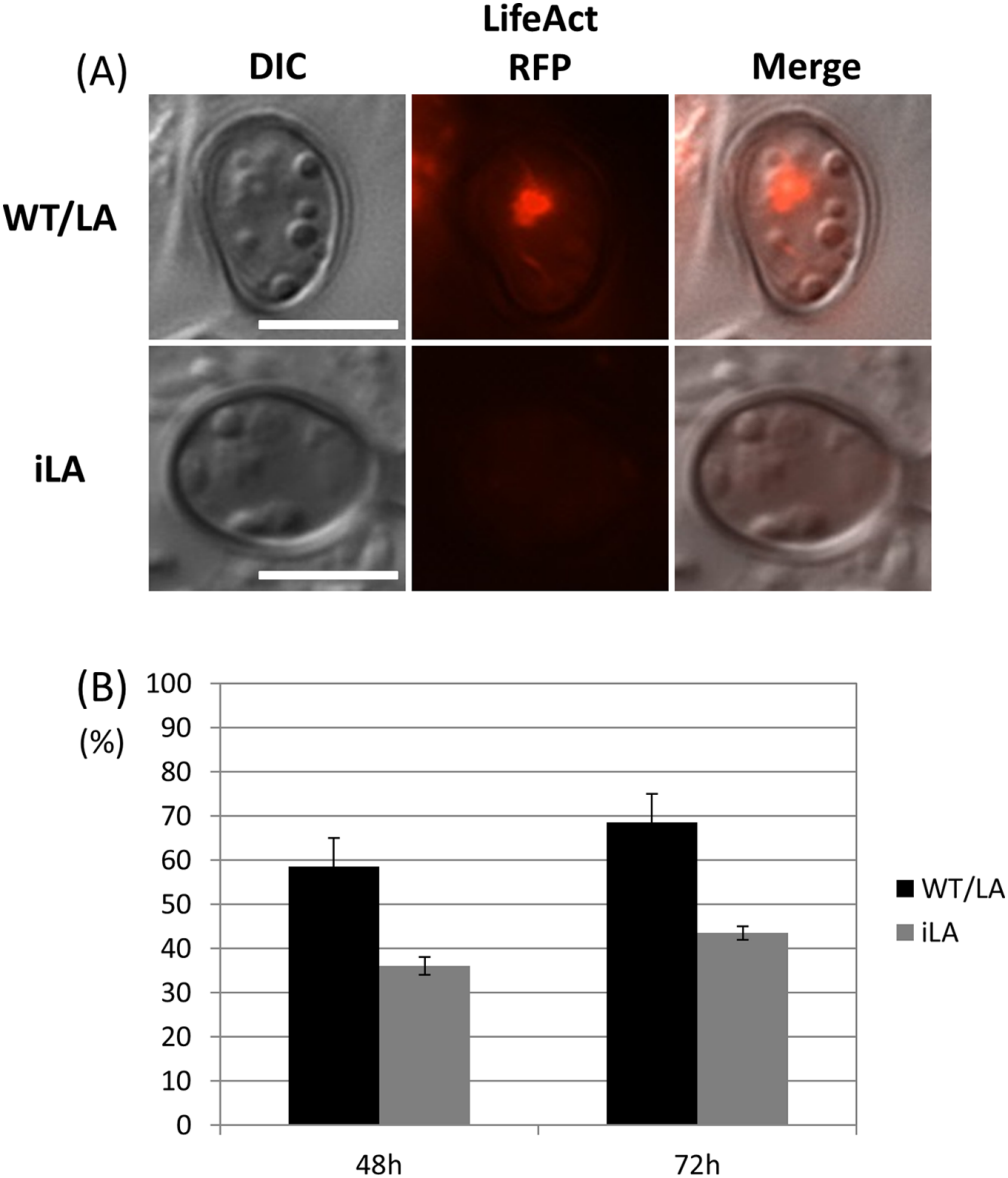
Assembly of F-actin in the appressorium pores of the *coira1* mutant. (A) Micrograph of F-actin organization in the appressorium pore visualized by the expression of Lifeact–RFP in the wild-type and in the *coira1* mutant. Conidial suspensions (10 µl) of each strain were applied to the abaxial surface of the cucumber cotyledons and incubated at 24°C for 48 h. WT/RA, the wild type expressing Lifeact–RFP; iRA, the *coira1* mutant expressing Lifeact–RFP (B) Percentage of the assembly of F-actin in the appressorium pore of the *coira1* mutants on the abaxial surface of cucumber cotyledons. Approximately 100 appressoria of each strain were observed per incubated site for 48 h, 72 h post-inoculation. Two replicates were examined. Three independent experiments were conducted, and standard errors are shown. Black bar, WT/RA; gray bar, Ira; WT/RA, the wild type expressing LifeAct-RFP; iRA, the *coira1* mutant expressing LifeAct-RFP.

In *M. oryzae*, it has been reported that proper assembly of the F-actin network in the appressorium pore is required for host infection and MAPK *Mst12*, the *C. orbiculare Cst1* homolog, is known to be involved in the proper assembly of the F-actin network in the appressorium pore [37], [38]. Moreover, it has been reported that cAMP signaling is involved in the remodeling of the actin structure in *S. cerevisiae* [39]. Our data showed that CoIra1 regulated the cAMP and MAPK signaling pathways (Figure 5 and 7). To elucidate whether CoIra1 colocalize with F-actin, we generated the LA/IRA1–VENUS transformant by introducing Lifeact–*RFP* into it. In *C. orbiculare*, Lifeact, which binds to F-actin, forms vesicle-like structures in pregerminated conidia and then Lifeact–*RFP* mainly localizes in the appressorium pore during appressoria development. We examined the localization of CoIra1–VENUS and Lifeact–*RFP* in pregerminated conidia and appressorium pores. CoIra1–VENUS was colocalized with Lifeact–*RFP* in pregerminated conidia, however, CoIra1–VENUS was not clearly colocalized with Lifeact–RFP in the appressorium pore (Figure 13). To elucidate whether CoIra1 is involved in the assembly of the F-actin in the appressorium pore, we generated the iLA transformant by introducing Lifeact–*RFP* into the *coira1* mutant. In *C. orbiculare*, the wild-type showed a specific assembly of F-actin in the appressorium pore on the cucumber cotyledons, however, the frequency of this assembly in the *coira1* mutant was lower than that in the wild-type (Figure 14). These data indicated that CoIra1 is involved in the assembly of the F-actin in the appressorium pore.

## Discussion

### CoIra1 is involved in the crosstalk between cAMP and MAPK signaling pathways through CoRas2 in *C. orbiculare*

**Figure 15:**
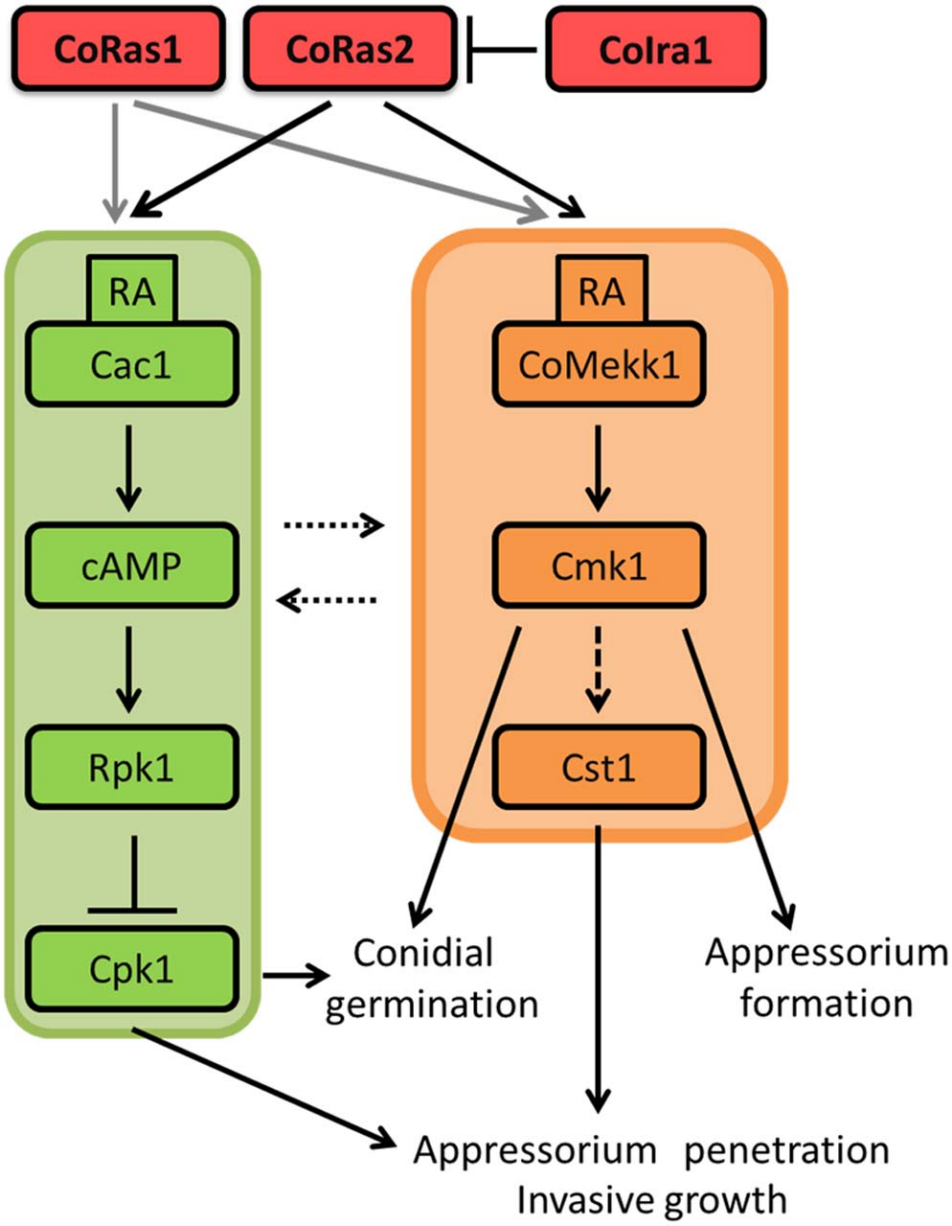
The hypothetical model for CoIra1 and CoRas1/2 mediated signaling transduction pathway in *C. orbiculare*. The cAMP-PKA signaling pathway is involved in conidial germination, appressorium penetration and invasive hyphae formation. The MAPK CoMekk1–Cmk1 signaling pathway is involved in conidial germination and appressorium formation. The CoRas2 localization pattern in the *coira1* mutant was similar to that in DARS2. Moreover, BiFC assays supported that CoIra1 interacted with CoRas2 in the plasma membrane. Therefore, CoIra1 negatively regulates CoRas2. The *coira1* mutant and DARS2 significantly induced abnormal appressorium formation and the frequency of abnormal appressorium formation in iDNRS2 was lower compared with that in the *coira1* mutant. Moreover, intracellular cAMP levels in the *coira1* mutant and DARS2 was high compared with those in the wild type. Therefore, CoIra1 regulates cAMP-PKA signaling pathway through CoRas2. The conidia of the *coras2* mutant failed to germinate, whereas DRS2/DAMK1 restored the phenotype of the *coras2* mutant. Therefore, CoRas2 is an upstream regulator of the MAPK CoMekk1–Cmk1 signaling pathway. Interestingly, the phosphorylation of MAPK Cmk1 in the *coras2* mutant was higher compared with that in the wild-type, although CoRas2 is an upstream regulator of CoMekk1–Cmk1. Therefore, that CoIra1 may be a key factor for regulating the crosstalk between the cAMP–PKA and CoMekk1–Cmk1 MAPK signaling pathway through CoRas2. Intracellular cAMP levels in DARS1 were higher compared with those in the wild-type in vegetative hyphae. However, DARS1 showed lower sensitivity to exogenous cAMP in appressorium development compared with DARS2. Moreover, the intensity of pathogenesis in DARS1 was similar to that in the wild-type. Therefore, CoRas1 could be involved in the cAMP–PKA signaling pathway in vegetative hyphae but not during infection related morphogenesis.

In *S. cerevisiae*, the activation of the cAMP-dependent pathway causes cells to undergo unipolar growth, which is coupled with an elongated growth that is controlled by the filamentous MAPK pathway [40]. In *C. orbiculare*, cAMP–PKA signal transduction is involved in conidial germination, and the MAPK cascade is involved in appressorium development [1]. In *S. cerevisiae*, an increase in the intracellular levels of RAS–GTP against RAS–GDP is observed in *ira1* and *ira2* mutants, activating various target effectors [41]. Our data indicated that intracellular cAMP levels and the phosphorylation of MAPK Cmk1 in the *coira1* mutant, DARS1, and DARS2 was higher than that in the wild-type. Interestingly, the phosphorylation of Cmk1 in the *coras2* mutant was higher compared with the wild-type, although CoRas2 is an upstream regulator of CoMekk1–Cmk1. In *M. oryzae*, the intracellular cAMP levels of the dominant active MEK *Mst7* ^S212D T216E^ strain are lower than those of the wild-type [42]. Recently, the functional analysis of the adenylate cyclase-associated protein encoding *CAP1*, the ortholog of yeast *Srv2*, Cap1 may play a role in the feedback inhibition of MoRas2 signaling when Pmk1 MAP kinase is activated [12]. In yeast and phytopathogenic fungi, cAMP signaling is intimately associated with a MAPK homologous with PMK1 for regulating various developmental and plant-infection processes [43]–[45]. We assumed that the constitutively active forms CoRas1 and CoRas2 induce excessive activation of the cAMP–PKA and MAPK CoMekk1–Cmk1 signaling pathways, resulting in the interruption of the cAMP–PKA and MAPK signaling pathways (Figure 15). Interestingly, the level of cAMP and the phosphorylation of Cmk1 in DARS1 was higher than that in the DARS2; however, the pathogenicity of DARS1 was not attenuated during the infection of cucumber leaves (Figure S11). Therefore, CoRas1 may be involved in the cAMP–PKA and MAPK CoMekk1–Cmk1 signaling pathways in vegetative hyphae. To understand Ras-mediated signaling pathways clearly, further functional analysis of the relationship between CoRas1 and CoRas2 during infection is required.

### CoIra1 interacts with CoRas2 and negatively regulates CoRas2

Ras serves as a molecular switch, coupling activated membrane receptors to the downstream signaling molecule, by alternating between the GTP-bound (active) and the GDP bound (inactive) conformations. In *S. cerevisiae*, Ira1/2 negatively regulates Ras1/2; therefore, the ira1/2 mutants accumulate high amounts of Ras–GTP compared with the wild-type [7]. In the present study, the localization pattern of CoRas2 in the *coira1* mutant was similar to that in the active form CoRas2 in vegetative hyphae. Moreover, BiFC assays supported that CoIra1 regulates CoRas2 at the plasma membrane of vegetative hyphae. It is likely that CoIra1 negatively regulates CoRas2 in vegetative hyphae. In *Aspergillus fumigatus*, RasA localizes in plasma membranes in hyphae where it associates with and stimulates targeted effectors, resulting in the regulation of polarized morphogenesis [46]. We considered that CoIra1 negatively regulates CoRas2, which is required for the proper morphogenesis of vegetative hyphae. However, this morphogenesis in the *coira1* mutant and the dominant active form CoRas2 expressed strain was similar to that in the wild-type. In *C. orbiculare*, the conidia in the MAPK *cmk1* disruption mutant fails to germinate, however, by adding 0.1-% yeast extract, conidial germination efficiency can be restored [33]. Thus, there is a strong possibility that a nutrient-specific signaling pathway regulates conidial germination in *C. orbiculare*. Therefore, we speculated the presence of a bypass pathway that regulates vegetative hyphae morphogenesis independent of the CoIra1–CoRas2 signaling pathway in the presence of nutrients.

### *CoIRA1* regulates proper infection-related morphogenesis

Our data indicated that the pathogenicity of the *coira1* mutant was attenuated during the infection of the cucumber cotyledons. Plants have evolved with a variety of defense mechanisms against attacking phytopathogenic fungi. These include the deposition of cell wall reinforcement components (papillae), hypersensitive cell death, and the synthesis of an antimicrobial secondary metabolite. However, callose deposition was not observed following attempted penetration from the appressoria of the *coira1* mutant. Furthermore, the pathogenesis of the *coira1* mutant was not restored during the infection of heat-shocked cucumber cotyledons. Therefore, there is a small possibility that the observed reduction of pathogenicity in the *coira1* mutant was caused by the plant defense response. In *C. orbiculare*, the generation of the appressorium turgor pressure is required for mechanical penetration of the plant surface [47], however, appressorial turgor of the *coira1* mutant was not affected, as shown by the cytorrhysis assay [27]. The pathogenicity of the *coira1* mutant was attenuated compared with that of the wild-type during infection of wounded leaves, thereby resulting in the reduction in the development of hyphae causing infection in the host plants. In *C. orbiculare*, the catalytic subunit of PKA Cpk1 and adenylate cyclase Cac1 is involved in infectious growth in plants [3]. We postulate that excessive cAMP levels in the *coira1* mutant would affect the process of the infection hyphae development in the host plant. The vegetative hyphae of the *coira1* mutant and the wild-type showed normal morphology; however, the *coira1* mutant formed bulb-shaped hyphae on the cellulose membranes. In response to nitrogen starvation, diploid *S. cerevisiae* cells undergo pseudohyphal growth, which is enhanced by the expression of the dominant active allele *RAS2* [48]. Further investigation has revealed that the pseudohyphal growth is controlled by both Kss1 MAPK and cAMP–PKA signaling [49]. Similarly, *C. albicans* strains carrying the activating *RAS*1^V13^ allele formed more abundant hyphae in a shorter time than that in the wild-type strain. Thus, activated Ras is a key factor for regulating hyphal morphogenesis in fungi. In this study, the molecular mechanism of bulb-shape penetration hyphae caused by the *coira1* mutation remains unclear. Therefore, further functional analysis of CoIra1 will be helpful for understanding the regulation of penetration hyphae morphogenesis for the requirement of proper infection of the host plant.

### CoIra1 and CoRas2 is involved in the appressoria-mediated differentiation of infection hyphae development

CoRas2 and CoIra1 did not show specific colocalization in developing appressoria. Consistently, CoIra1 and the active form CoRas2 did not colocalize in developing appressoria. Therefore, during appressorium formation, CoRas2 may be regulated by other factors different from CoIra1. In *C. orbiculare*, 48 to 72 h post-inoculation is a crucial phase for appressorium-mediated penetration. Our data indicated that CoIra1 and CoRas2 colocalized in a vesicle-like structure in the appressoria 48 h post-inoculation. Moreover, in *M. oryzae*, the correct organization of F-actin in the appressorium pore is important for penetration peg development [37]. Our data also indicated that CoIra1 is involved in the assembly of the F-actin in the appressorium pore. Therefore, we assume that CoIra1 could be involved in the F-actin organization in appressoria, which is required for penetration peg emergence. However, it is likely that CoIra1 does not directly regulate F-actin, because it does not colocalize with the F-actin in the appressoria pore. In *M. oryzae*, NADPH oxidases regulate the septin-mediated assembly of F-actin in the appressorium pore [50]. Moreover, in *C. neoformans*, the GTP-bound form of Ras1 interacts with Rho–GEF Cdc24 that mediates the activation of Cdc42 and Rac proteins, and Cdc42 is involved in cytokinesis and bud morphogenesis through the organization of septin proteins [51]. In *Epichloë festucae*, NoxA is activated by a small GTPase RacA [52]. Therefore, in *C. orbiculare*, Ras–Cdc42–septin proteins and the Ras–Rac–Nox proteins signaling pathway may regulate the assembly of F-actin in the appressorium pore. Our future work will identify interaction factors between CoIra1 and CoRas2, elucidating those that are involved in the assembly of F-actin in the appressorial pore.

## Supporting Information

Figure S1 **Organization of the** ***CoIRA1*** **gene in** ***C. orbiculare*** **.** (A) Schematic representation of *CoIRA1*. Exons are indicated by gray boxes. The predicted RASGAP domains are indicated by slashed boxes. Eleven exons of *CoIRA1* are indicated by a gray square. Ten introns of *CoIRA1* are indicated by a black bar among 11 exons. (B) RASGAP domain in CoIra1. Amino acid sequence alignment of the predicted *CoIRA1* gene product with homologs from *Saccharomyces cerevisiae IRA1*, *IRA2*, and *Magnaporthe oryzae* (M.o.). Identical amino acids are indicated by a black background, similar residues are indicated by a gray background, and gaps introduced for alignments are indicated by a hyphen. The predicted RASGAP domain is indicated by a black line.
(TIF)

Figure S2 **Gene disruption of the** ***CoIRA1*** **of** ***C. orbiculare*** **.** (A) *CoIRA1* gene disruption by homologous recombination with the *CoIRA1* disruption vector in which a *hph* fragment was inserted into the *CoIRA1* gene. Through double crossover, an *Eco*RV fragment of approximately 3.4 kb containing wild-type *CoIRA1* was predicted to be replaced by a fragment of approximately 5.6 kb containing the *hph* fragment. (B) *CoIRA1* gene disruption was confirmed by Southern blot analysis. Genomic DNAs from the wild-type 104-T and transformants were digested with *Eco*RV and probe with an upstream 1.0-kb fragment of the *CoIRA1* gene. WT, wild-type; dis1-5, *coira1* mutants; ec1-2, ectopic strains.
(TIF)

Figure S3 **Hyphal growth and conidia number of the** ***coira1*** **mutant on PDA.** (A) Each strain was grown on the PDA medium at 24°C for five days and the number of conidia harvested from a 9-cm PDA plate at 5 days after incubation at 24°C.
(TIF)

Figure S4 **Appressorium cytorrhysis assay for the** ***coira1*** **mutant.** Appressoria formed on the multiwell slides were exposed to glycerol solutions ranging from 0 M to 4 M and the percentage of collapsed spherical appressorium was counted. Approximately 200 conidia were observed per well. Three replicates were examined. Three independent experiments were conducted, and standard errors are shown. Solid line, the wild-type; dotted line, the *coira1* mutant.
(TIF)

Figure S5 **Pathogenicity assay of the** ***coira1*** **mutant in wounded leaves.** Conidial suspensions of each strain were inoculated on wounded sites on the cotyledon of the cucumber prepared by scratching the leaves with a sterile toothpick. The leaves were incubated at 24°C for seven days. Strains: WT, wild-type 104-T; DL1, the *coira1* mutant; CL1, the *CoIRA1*-complemented transformant of DL1; DPS1, the *pks1* mutant.
(TIF)

Figure S6 **Host defense response was not induced by the penetration of the** ***coira1*** **mutant.** (A) Quantification of papilla formation at sites of attempted penetration by appressorium in *C. orbiculare.* At three days, leaf epidermal strips inoculated with each strain was stained with Aniline blue to reveal the papilla and observed with epi-fluorescence microscopy. Strains: WT, wild-type; DL1, the *coira1* mutant; CL1, the *CoIRA1*-complemented transformant of DL1; DSD1, the *ssd1* mutant. At least 200 appressoria were counted for each strain and standard deviations were calculated from three replicated experiments. (B) Pathogenicity assay of the *coira1* mutant on heat-shock cotyledons after the heat treatment at 50°C for 30 s, cucumber cotyledons were inoculated with conidial suspensions. Strains: WT, wild-type; DL1, *coira1* mutant; CL1, the *CoIRA1*-complemented transformant of DL1; DSD1, the *ssd1* mutant. Controls were not exposed to heat shock.
(TIF)

Figure S7 **Penetration hyphae formation of a dominant active form** ***CoRAS1*** **and** ***CoRAS2*** **introduced transformants on cellulose membranes.** Conidial suspensions of each strain in distilled water were incubated on cellulose membranes at 24°C for 48 h. WT, the wild-type 104-T; DARS1, WT transformed with a dominant active form *CoRAS1*; DARS2, WT transformed with a dominant active form *CoRAS2.* Scale bar, 10 µm. (B) Percentages of penetration hyphae formation, and bulb-shaped penetration-hyphae formation of *C. orbiculare* WT, DARS1 and DARS2 on cellulose membranes. Approximately 200 conidia of each strain were observed on cellulose membranes. Three replicates were examined. Three independent experiments were conducted, and standard errors are shown. black bar, penetration hyphae; gray bar, bulb-shape penetration hyphae formation.
(TIF)

Figure S8 **Pathogenicity assay of a dominant active form** ***CoRAS1*** **and** ***CoRAS2*** **introduced** **transformants on cucumber cotyledons.** Conidial suspensions of each strain were inoculated on the detached cucumber cotyledons, and the leaves were incubated at 24°C for seven days. WT, the wild-type; DARS1, WT transformed with a dominant active form *CoRAS1*; DARS2, WT transformed with a dominant active form *CoRAS2.*
(TIF)

Figure S9 **Appressorium formation in** ***coira1*** **mutants of** ***C. orbiculare*** **on glass slides.** (A) Conidial suspensions of each strain in distilled water were incubated on multiwell glass slides at 24°C for 24 h. WT, wild-type; DRS2-1 and DRS2-2, the *coras2* mutant; CRS2-1, the *CoRAS2*-complemented transformant of DRS2-1; CR2-2, the *CoRAS2*-complemented transformant of DRS2-2. Scale bar, 10 µm. (B) Percentages of conidial germination, appressorium formation in *C. orbiculare* WT and *coras2* mutants on multiwell glass slides. Approximately 100 conidia of each strain were observed per well on multiwell glass slides. Three replicates were examined. Three independent experiments were conducted, and standard errors are shown. Black bar, conidial germination; gray bar, appressorium formation.
(TIF)

Figure S10 **Pathogenicity assay of** ***coras2*** **mutants of** ***C. orbiculare*** **on the cucumber cotyledons.** Conidial suspensions of each strain were placed on detached cotyledons of the cucumber, and the leaves were incubated at 24°C for seven days. Shown are the leaves after incubation with the following strains: WT, wild-type 104-T; DRS2-1 and DSR2-2, the *coras2* mutant; CRS2-1, the *CoRAS2*-complemented transformant of DRS2-1; the CRS2-2, *CoRAS2*-complemented transformant of DRS2-2.
(TIF)

Figure S11 **Assay for colocalizations of CoIra1 and active form CoRas2.** Conidial suspensions of RFP–DARS2/IRA1–VENUS strain were incubated on glass slides at 24°C for 0 h, 3 h, 6 h and 24 h and observed by fluorescent microscopy. RFP–DARS2/IRA1–VENUS, the wild-type strain expressing RFP fused with a dominant active form *CoRAS2* and *CoIRA1*–VENUS. Scale bar, 10 µm.
(TIF)

Figure S12 **Localization of a functional CoIra1–VENUS fusion protein in** ***C. orbiculare*** **in initial and late infection hyphae in the cucumber leaves.** The wild-type strain expressing *CoIRA1*–VENUS was inoculated on cucumber leaves and incubated at 48 h, 72 h and CoIra1–VENUS was observed using fluorescent microscopy.
(TIF)

Table S1 PCR primers used in this study.
(TIF)
