# Supplementary figures and images for: Ras GTPase Activating Protein CoIra1 Is Involved in Infection-Related Morphogenesis by Regulating cAMP and MAPK Signaling Pathways through CoRas2 in Colletotrichum orbiculare
Source: PLoS One. 2014 Oct 2;9(10):e109045. doi: 10.1371/journal.pone.0109045 (PMC4183519; doi:10.1371/journal.pone.0109045)

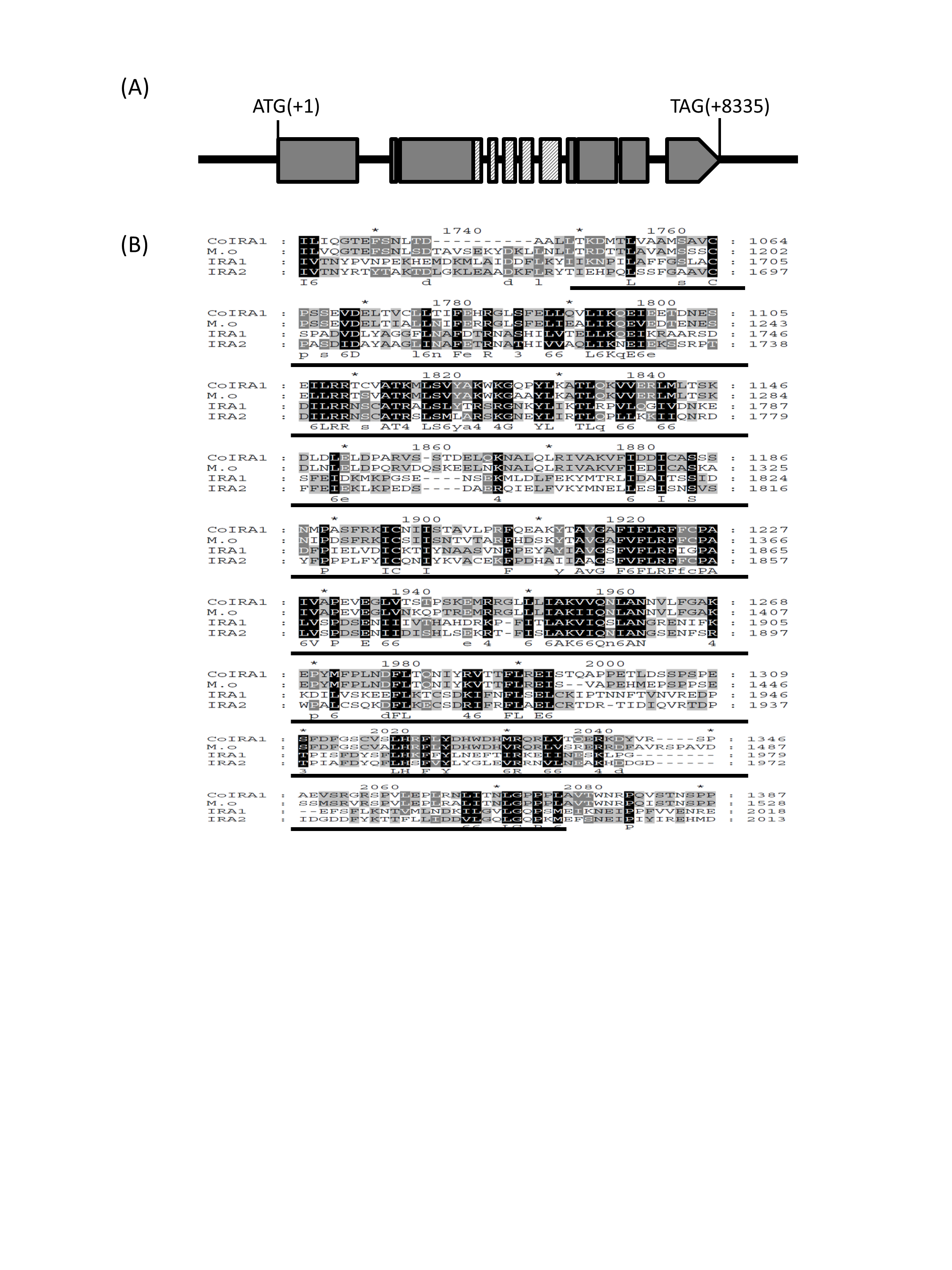

Supplement: Figure S1 — Organization of the CoIRA1 gene in C. orbiculare . (A) Schematic representation of CoIRA1. Exons are indicated by gray boxes. The predicted RASGAP domains are indicated by slashed boxes. Eleven exons of CoIRA1 are indicated by a gray square. Ten introns of CoIRA1 are indicated by a black bar among 11 exons. (B) RASGAP domain in CoIra1. Amino acid sequence alignment of the predicted CoIRA1 gene product with homologs from Saccharomyces cerevisiae IRA1, IRA2, and Magnaporthe oryzae (M.o.). Identical amino acids are indicated by a black background, similar residues are indicated by a gray background, and gaps introduced for alignments are indicated by a hyphen. The predicted RASGAP domain is indicated by a black line. (TIF) [file pone.0109045.s001.tif]

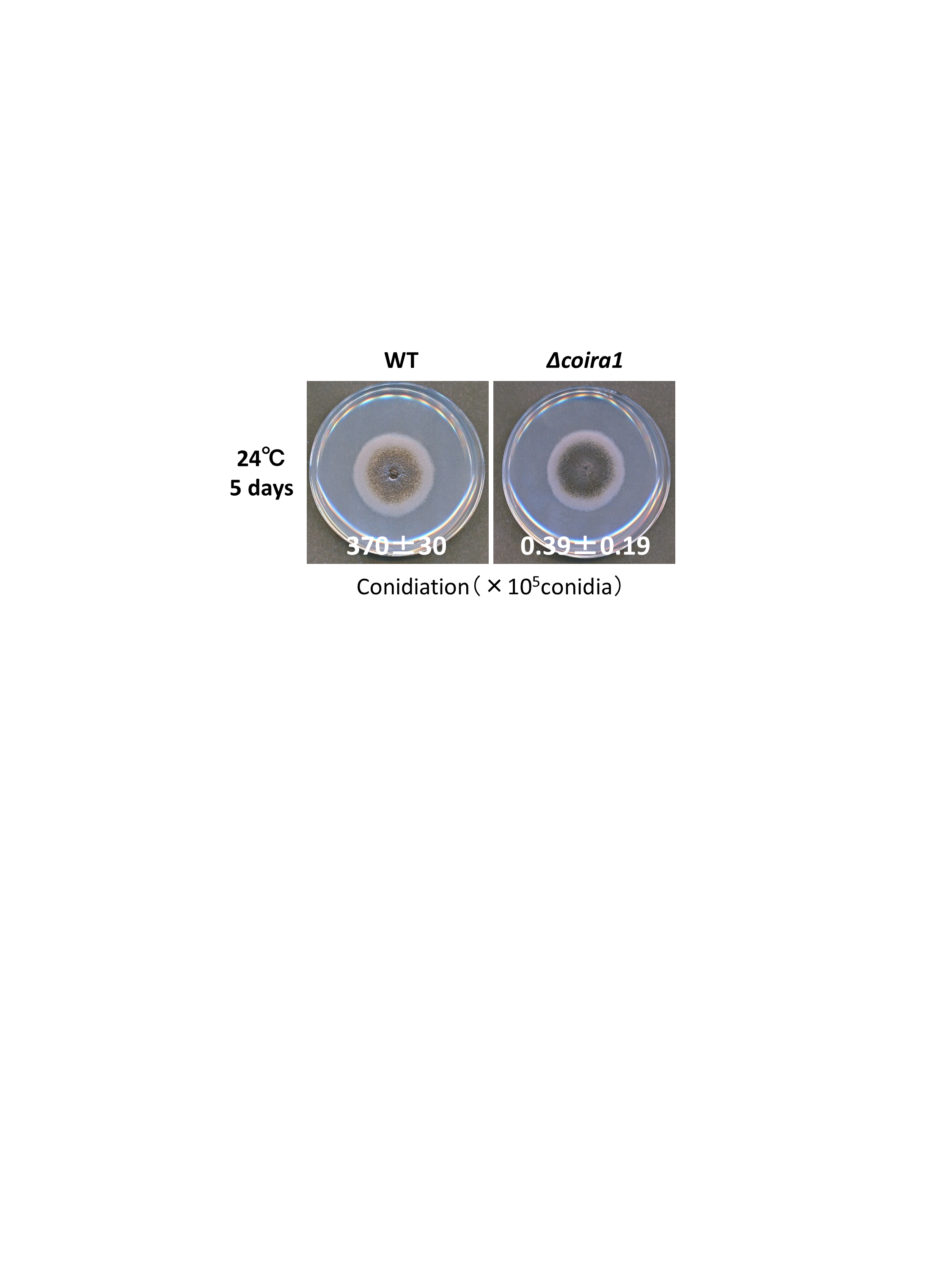

Supplement: Figure S3 — Hyphal growth and conidia number of the coira1 mutant on PDA. (A) Each strain was grown on the PDA medium at 24°C for five days and the number of conidia harvested from a 9-cm PDA plate at 5 days after incubation at 24°C. (TIF) [file pone.0109045.s003.tif]

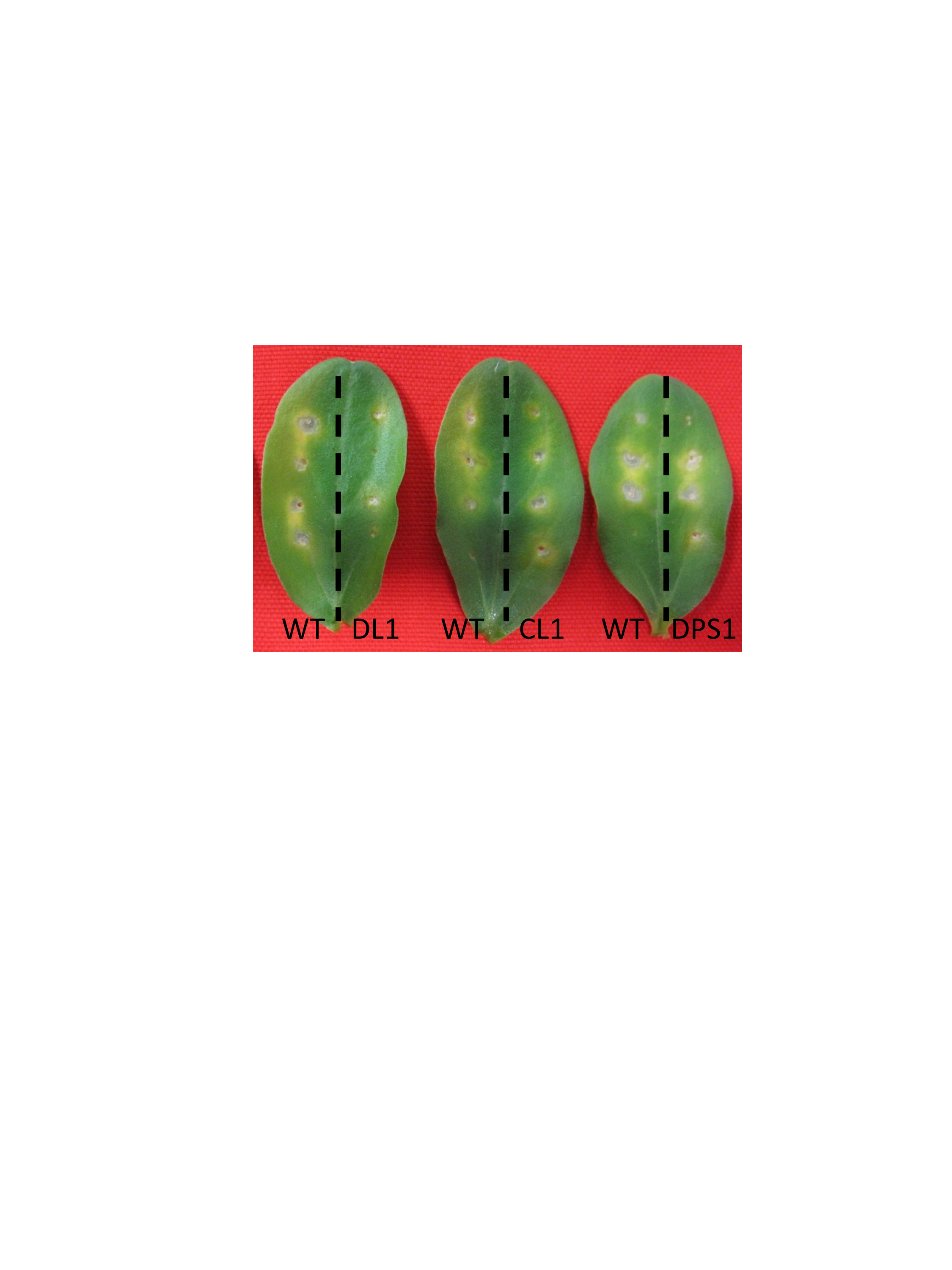

Supplement: Figure S5 — Pathogenicity assay of the coira1 mutant in wounded leaves. Conidial suspensions of each strain were inoculated on wounded sites on the cotyledon of the cucumber prepared by scratching the leaves with a sterile toothpick. The leaves were incubated at 24°C for seven days. Strains: WT, wild-type 104-T; DL1, the coira1 mutant; CL1, the CoIRA1-complemented transformant of DL1; DPS1, the pks1 mutant. (TIF) [file pone.0109045.s005.tif]

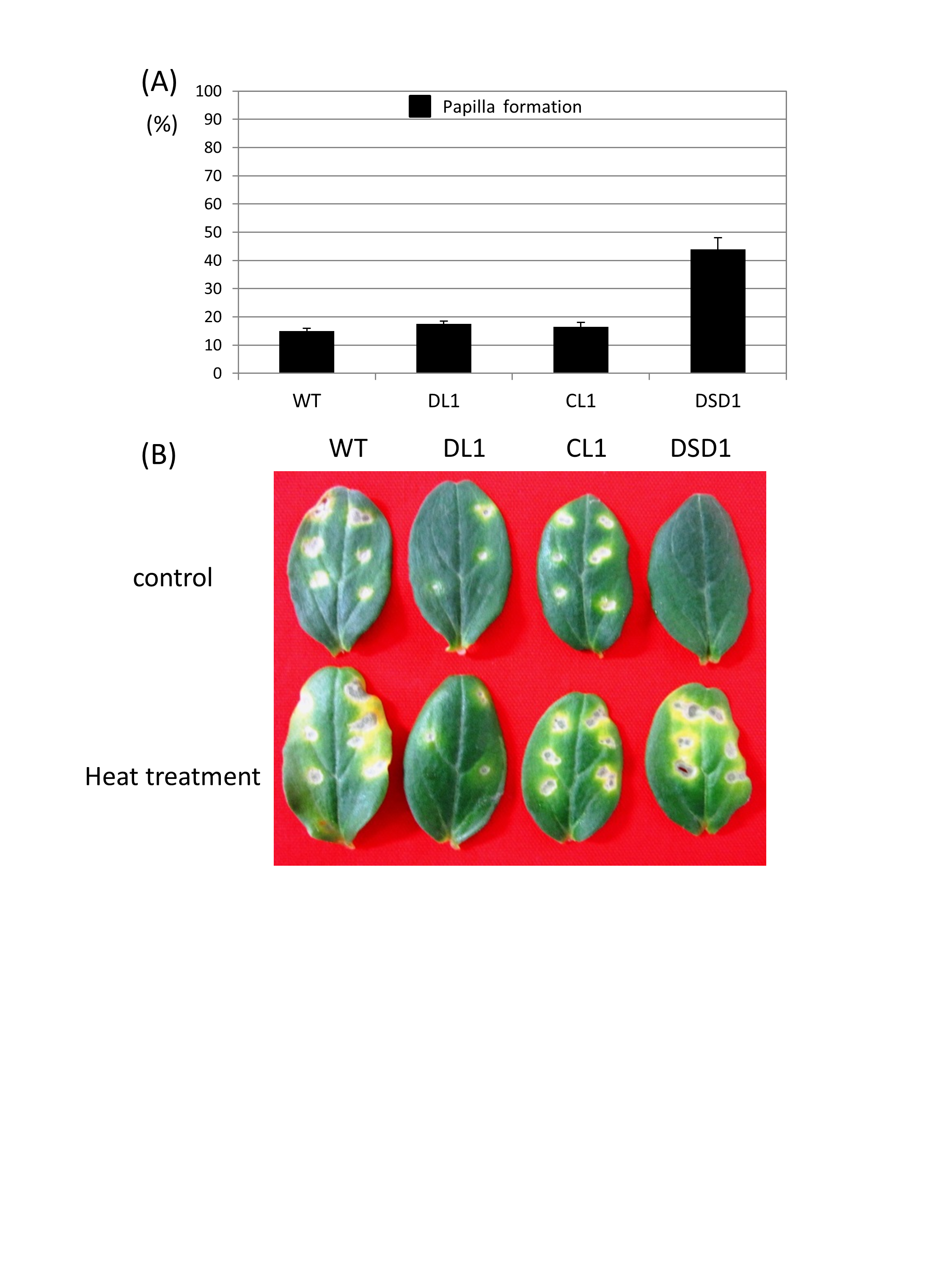

Supplement: Figure S6 — Host defense response was not induced by the penetration of the coira1 mutant. (A) Quantification of papilla formation at sites of attempted penetration by appressorium in C. orbiculare. At three days, leaf epidermal strips inoculated with each strain was stained with Aniline blue to reveal the papilla and observed with epi-fluorescence microscopy. Strains: WT, wild-type; DL1, the coira1 mutant; CL1, the CoIRA1-complemented transformant of DL1; DSD1, the ssd1 mutant. At least 200 appressoria were counted for each strain and standard deviations were calculated from three replicated experiments. (B) Pathogenicity assay of the coira1 mutant on heat-shock cotyledons after the heat treatment at 50°C for 30 s, cucumber cotyledons were inoculated with conidial suspensions. Strains: WT, wild-type; DL1, coira1 mutant; CL1, the CoIRA1-complemented transformant of DL1; DSD1, the ssd1 mutant. Controls were not exposed to heat shock. (TIF) [file pone.0109045.s006.tif]

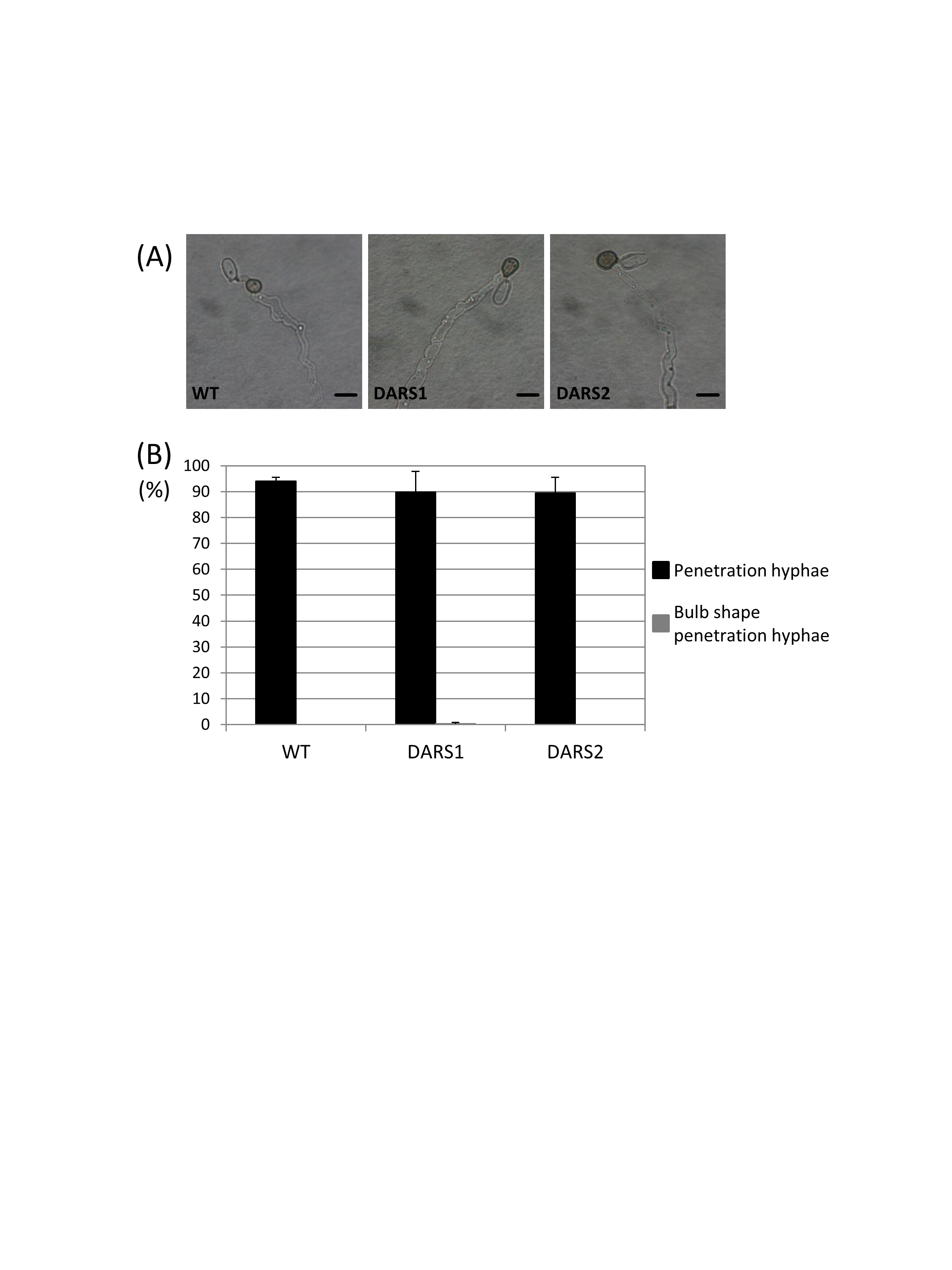

Supplement: Figure S7 — Penetration hyphae formation of a dominant active form CoRAS1 and CoRAS2 introduced transformants on cellulose membranes. Conidial suspensions of each strain in distilled water were incubated on cellulose membranes at 24°C for 48 h. WT, the wild-type 104-T; DARS1, WT transformed with a dominant active form CoRAS1; DARS2, WT transformed with a dominant active form CoRAS2. Scale bar, 10 µm. (B) Percentages of penetration hyphae formation, and bulb-shaped penetration-hyphae formation of C. orbiculare WT, DARS1 and DARS2 on cellulose membranes. Approximately 200 conidia of each strain were observed on cellulose membranes. Three replicates were examined. Three independent experiments were conducted, and standard errors are shown. black bar, penetration hyphae; gray bar, bulb-shape penetration hyphae formation. (TIF) [file pone.0109045.s007.tif]

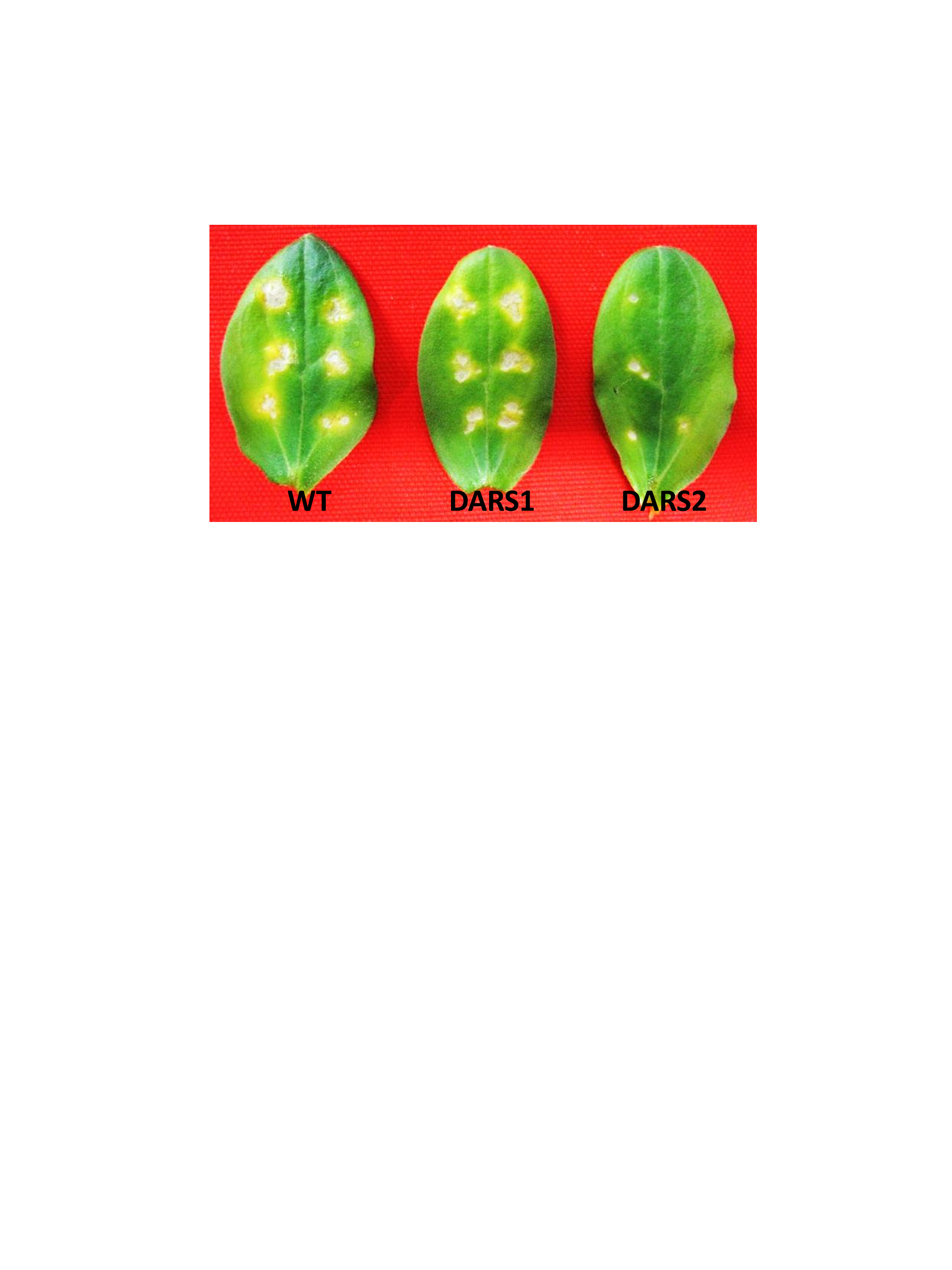

Supplement: Figure S8 — Pathogenicity assay of a dominant active form CoRAS1 and CoRAS2 introduced transformants on cucumber cotyledons. Conidial suspensions of each strain were inoculated on the detached cucumber cotyledons, and the leaves were incubated at 24°C for seven days. WT, the wild-type; DARS1, WT transformed with a dominant active form CoRAS1; DARS2, WT transformed with a dominant active form CoRAS2. (TIF) [file pone.0109045.s008.tif]

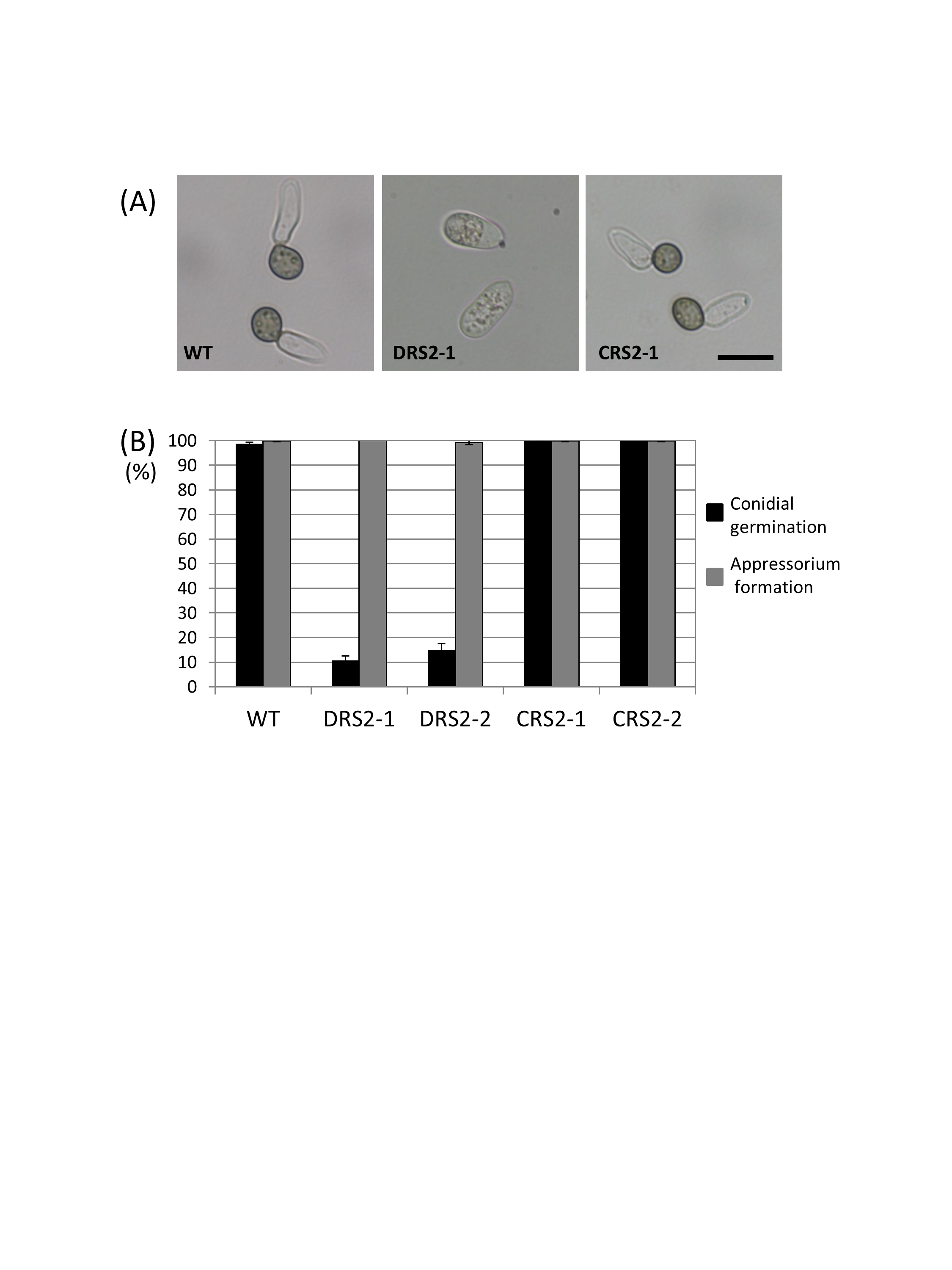

Supplement: Figure S9 — Appressorium formation in coira1 mutants of C. orbiculare on glass slides. (A) Conidial suspensions of each strain in distilled water were incubated on multiwell glass slides at 24°C for 24 h. WT, wild-type; DRS2-1 and DRS2-2, the coras2 mutant; CRS2-1, the CoRAS2-complemented transformant of DRS2-1; CR2-2, the CoRAS2-complemented transformant of DRS2-2. Scale bar, 10 µm. (B) Percentages of conidial germination, appressorium formation in C. orbiculare WT and coras2 mutants on multiwell glass slides. Approximately 100 conidia of each strain were observed per well on multiwell glass slides. Three replicates were examined. Three independent experiments were conducted, and standard errors are shown. Black bar, conidial germination; gray bar, appressorium formation. (TIF) [file pone.0109045.s009.tif]

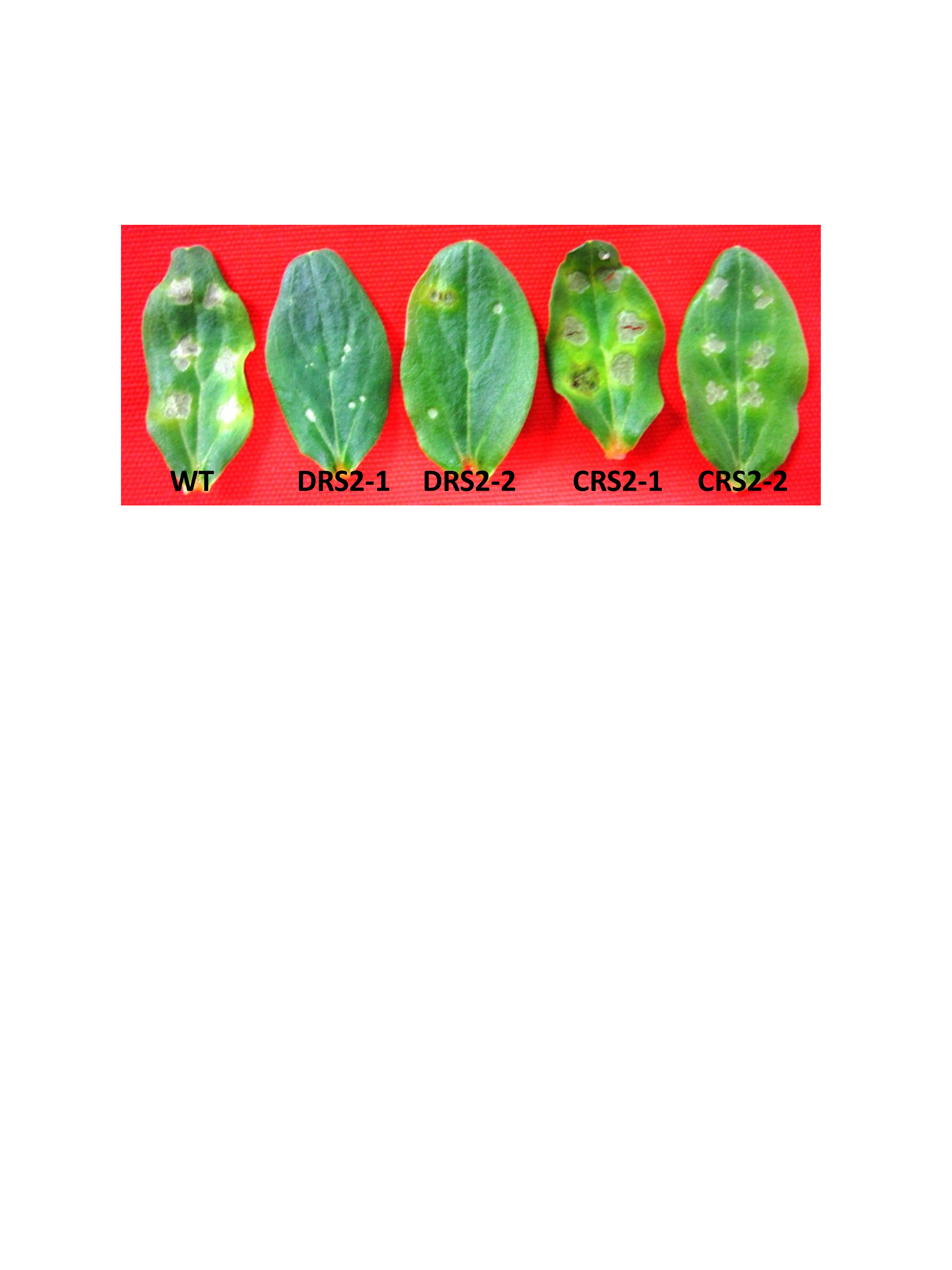

Supplement: Figure S10 — Pathogenicity assay of coras2 mutants of C. orbiculare on the cucumber cotyledons. Conidial suspensions of each strain were placed on detached cotyledons of the cucumber, and the leaves were incubated at 24°C for seven days. Shown are the leaves after incubation with the following strains: WT, wild-type 104-T; DRS2-1 and DSR2-2, the coras2 mutant; CRS2-1, the CoRAS2-complemented transformant of DRS2-1; the CRS2-2, CoRAS2-complemented transformant of DRS2-2. (TIF) [file pone.0109045.s010.tif]

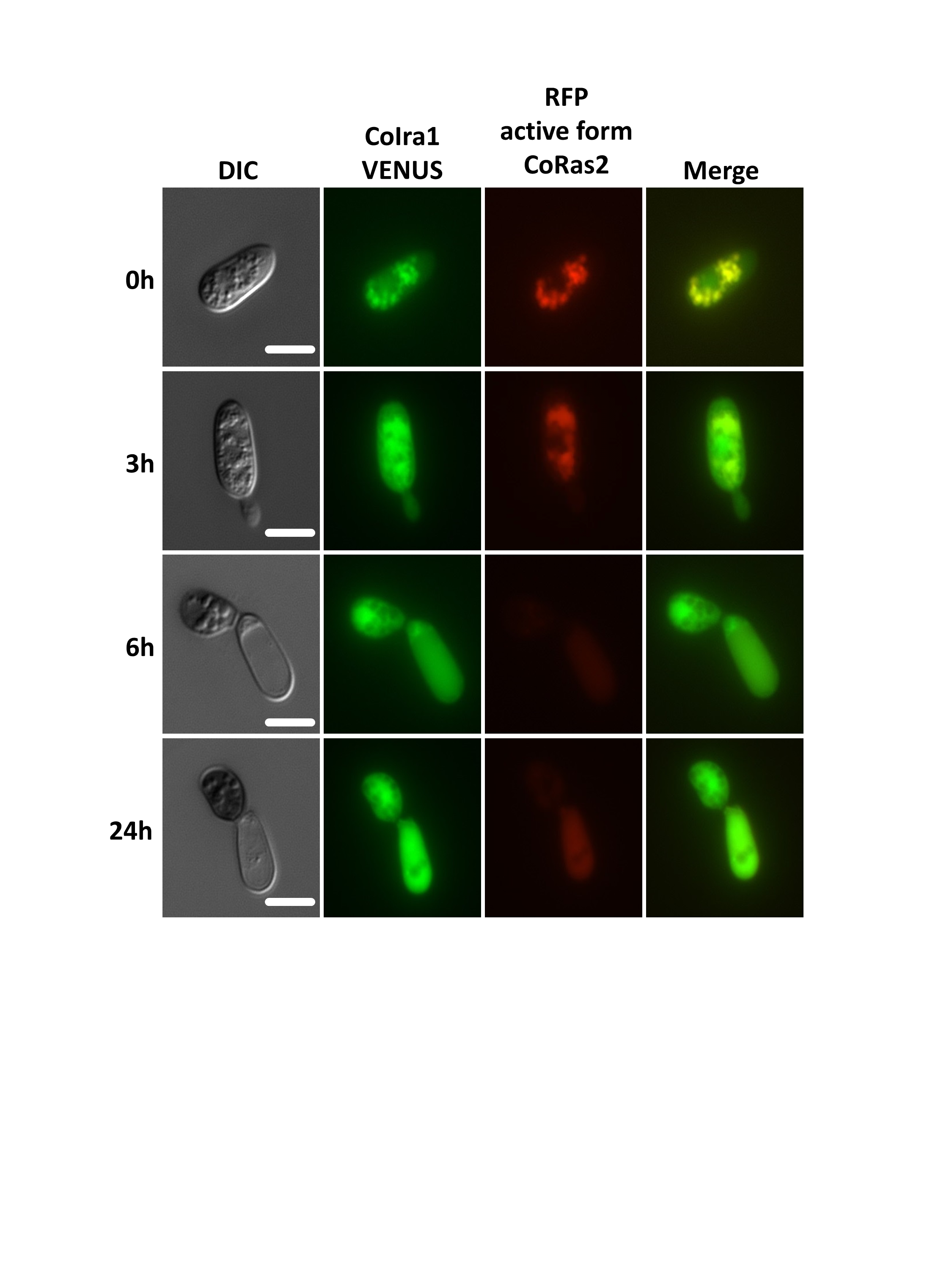

Supplement: Figure S11 — Assay for colocalizations of CoIra1 and active form CoRas2. Conidial suspensions of RFP–DARS2/IRA1–VENUS strain were incubated on glass slides at 24°C for 0 h, 3 h, 6 h and 24 h and observed by fluorescent microscopy. RFP–DARS2/IRA1–VENUS, the wild-type strain expressing RFP fused with a dominant active form CoRAS2 and CoIRA1–VENUS. Scale bar, 10 µm. (TIF) [file pone.0109045.s011.tif]

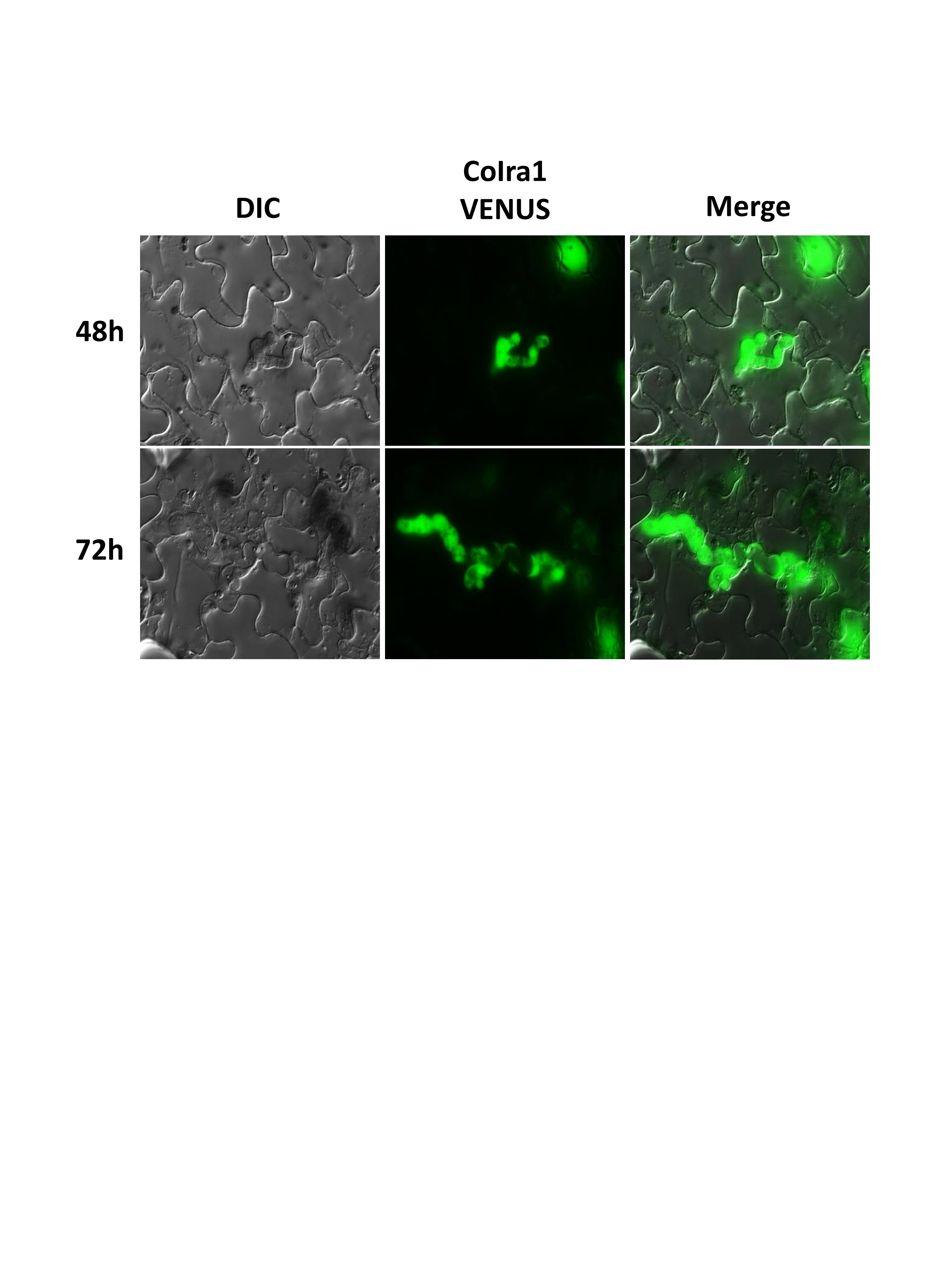

Supplement: Figure S12 — Localization of a functional CoIra1–VENUS fusion protein in C. orbiculare in initial and late infection hyphae in the cucumber leaves. The wild-type strain expressing CoIRA1–VENUS was inoculated on cucumber leaves and incubated at 48 h, 72 h and CoIra1–VENUS was observed using fluorescent microscopy. (TIF) [file pone.0109045.s012.tif]
